# Trends in the Prevalence of Metabolically Healthy Obesity Among US Adults, 1999-2018

**DOI:** 10.1001/jamanetworkopen.2023.2145

**Published:** 2023-03-09

**Authors:** Jiang-Shui Wang, Peng-Fei Xia, Meng-Nan Ma, Yue Li, Ting-Ting Geng, Yan-Bo Zhang, Zhou-Zheng Tu, Limiao Jiang, Li-Rong Zhou, Bing-Fei Zhang, Wen-Wei Tong, Zhilei Shan, Gang Liu, Kun Yang, An Pan

**Affiliations:** 1 Ministry of Education Key Laboratory of Environment and Health, Department of Epidemiology and Biostatistics, School of Public Health, Tongji Medical College, Huazhong University of Science and Technology, Wuhan, Hubei Province, China; 2 Department of Endocrinology, Affiliated Dongfeng Hospital, Hubei University of Medicine, Shiyan, Hubei Province, China; 3 Hubei Key Laboratory of Food Nutrition and Safety, Department of Nutrition and Food Hygiene, School of Public Health, Tongji Medical College, Huazhong University of Science and Technology, Wuhan, Hubei Province, China

## Abstract

**Importance:**

Improved understanding of trends in the proportion of individuals with metabolically healthy obesity (MHO) may facilitate stratification and management of obesity and inform policy efforts.

**Objectives:**

To characterize trends in the prevalence of MHO among US adults with obesity, overall and by sociodemographic subgroups.

**Design, Setting, and Participants:**

This survey study included 20 430 adult participants from 10 National Health and Nutrition Examination Survey (NHANES) cycles between 1999-2000 and 2017-2018. The NHANES is a series of cross-sectional and nationally representative surveys of the US population conducted continuously in 2-year cycles. Data were analyzed from November 2021 to August 2022.

**Exposures:**

National Health and Nutrition Examination Survey cycles from 1999-2000 to 2017-2018.

**Main Outcomes and Measures:**

Metabolically healthy obesity was defined as a body mass index of 30.0 (calculated as weight in kilograms divided by height in meters squared) without any metabolic disorders in blood pressure, fasting plasma glucose (FPG), high-density lipoprotein cholesterol (HDL-C), or triglycerides based on established cutoffs. Trends in the age-standardized prevalence of MHO were estimated using logistic regression analysis.

**Results:**

This study included 20 430 participants. Their weighted mean (SE) age was 47.1 (0.2) years; 50.8% were women, and 68.8% self-reported their race and ethnicity as non-Hispanic White. The age-standardized prevalence (95% CI) of MHO increased from 3.2% (2.6%-3.8%) in the 1999-2002 cycles to 6.6% (5.3%-7.9%) in the 2015-2018 cycles (*P* < .001 for trend). There were 7386 adults with obesity. Their weighted mean (SE) age was 48.0 (0.3) years, and 53.5% were women. The age-standardized proportion (95% CI) of MHO among these 7386 adults increased from 10.6% (8.8%-12.5%) in the 1999-2002 cycles to 15.0% (12.4%-17.6%) in the 2015-2018 cycles (*P* = .02 for trend). Substantial increases in the proportion of MHO were observed for adults aged 60 years or older, men, non-Hispanic White individuals, and those with higher income, private insurance, or class I obesity. In addition, there were significant decreases in the age-standardized prevalence (95% CI) of elevated triglycerides (from 44.9% [40.9%-48.9%] to 29.0% [25.7%-32.4%]; *P* < .001 for trend) and reduced HDL-C (from 51.1% [47.6%-54.6%] to 39.6% [36.3%-43.0%]; *P* = .006 for trend). There was also a significant increase in elevated FPG (from 49.7% [95% CI, 46.3%-53.0%] to 58.0% [54.8%-61.3%]; *P* < .001 for trend) but no significant change in elevated blood pressure (from 57.3% [53.9%-60.7%] to 54.0% [50.9%-57.1%]; *P* = .28 for trend).

**Conclusions and Relevance:**

The findings of this cross-sectional study suggest that the age-standardized proportion of MHO increased among US adults from 1999 to 2018, but differences in trends existed across sociodemographic subgroups. Effective strategies are needed to improve metabolic health status and prevent obesity-related complications in adults with obesity.

## Key Points

**Question:**

Has the prevalence of metabolically healthy obesity (MHO) changed among US adults in the past 20 years?

**Findings:**

In this survey study of 20 430 adults using data from the 1999-2018 National Health and Nutrition Examination Survey cycles, the age-standardized prevalence of MHO increased significantly from 3% in 1999-2002 to 7% in 2015-2018; the proportion of MHO among adults with obesity also increased significantly from 11% to 15%. Disparities existed in trends across sociodemographic subgroups.

**Meaning:**

The results of this study suggest that the prevalence of MHO among US adults with obesity has increased significantly in the past 2 decades, with variations across sociodemographic subgroups.

## Teaser

This survey study uses data from the 1999-2018 National Health and Nutrition Examination Survey cycles to examine trends in the prevalence of metabolically healthy obesity among US adults.

## Introduction

The prevalence of obesity has increased substantially in the past 2 decades, reaching an epidemic level in the US.^1^ Obesity is associated with most cardiovascular risk factors, including metabolic syndrome (MetS), hypertension, type 2 diabetes, and dyslipidemia.^2^ However, large interindividual heterogeneity in the development of obesity-related complications has been suggested.^3^ Despite increased body fat, a subset of people with obesity do not have obesity-related cardiometabolic abnormalities; this is referred to as metabolically healthy obesity (MHO).^4,5,6,7,8,9^ Individuals with MHO have favorable metabolic profiles and thus relatively lower risk for adverse cardiovascular consequences of obesity compared with individuals with metabolically unhealthy obesity (MUO).^4,10^ Evidence suggests that weight management strategies are more effective among individuals with MUO compared with those with MHO,^11,12^ indicating the potential value of the concept of obesity phenotypes.

Previous studies have reported on the proportion of US adults with MHO; however, the estimated prevalence of MHO varies widely across studies, partly due to large discrepancies in definitions.^4,5,13,14,15,16^ Most studies have used body mass index (BMI) to define obesity status and MetS components to reflect metabolically healthy status, but the cutoff values and number of parameters vary considerably. In recent years, researchers have proposed a strict definition of MHO as the absence of all MetS components in individuals with obesity, based on the rationale that patients with known cardiometabolic risk factors cannot be regarded as healthy.^17,18^ Evidence from a meta-analysis^10^ and prospective studies^19,20,21^ supports the comparable cardiovascular risk of MHO under this definition to that of metabolically healthy individuals with normal weight. Furthermore, insulin resistance and low-grade chronic inflammation, which provide additional information on metabolic health, have also been suggested as potential markers to assess MHO status.^9,10,22^ In the context of the obesity epidemic, better understanding of trends in MHO may facilitate the stratification and treatment of patients with obesity and inform policy efforts. However, whether the proportion of MHO, defined by conventional risk factors and other surrogate markers, has changed over the past 2 decades is largely unknown for US adults.

In this study, we aimed to characterize trends in the prevalence of MHO among US adults with obesity from 1999 to 2018, overall and in key sociodemographic subgroups. Our secondary objective was to compare trends in MHO under several commonly used criteria.

## Methods

### Study Population

The National Health and Nutrition Examination Survey (NHANES) is a serial, cross-sectional, national survey with a complex, stratified, multistage probability design to monitor the health status of the civilian US population. The NHANES has been conducted continuously in 2-year cycles since 1999. Details of the NHANES are described elsewhere.^23^ The NHANES was approved by the research ethics review board of the US Centers for Disease Control and Prevention (CDC) National Center for Health Statistics, and written informed consent was obtained from all adult participants.^23^ The Institutional Review Board of Tongji Medical College determined that this study was exempt from review given the use of deidentified data. This study followed the American Association for Public Opinion Research (AAPOR) reporting guideline.

We used data from 10 NHANES cycles between 1999-2000 and 2017-2018. The response rate decreased from 76% in 1999-2000 to 49% in 2017-2018. We included nonpregnant adults aged 20 years or older in the fasting subsample, whose blood samples were obtained after an overnight fast of at least 8 hours (eTable 1 in Supplement 1). The fasting subsample was included because fasting glucose level is a key component of the MHO definition. Individuals who did not fulfill the fasting criteria or had missing values for BMI or metabolic parameters of interest were excluded.

### Data Collection

Information on participant age, sex, race and ethnicity, education, income, insurance status, medical history, and medication use was collected through household questionnaires. Race and ethnicity was not consistently reported in the NHANES (eg, Hispanic participants were not oversampled before 2007 and non-Hispanic Asian participants were not classified until 2011).^24^ For consistency over time, we categorized participants as self-reported Mexican American, non-Hispanic Black, non-Hispanic White, or other race and ethnicity (eg, non-Hispanic Asian or multiple). The family income-to-poverty ratio reflected annual family income relative to the federal poverty threshold and was used as a measure of income classified into 3 groups (≤100%, 101%-399%, and ≥400%).^25^

Weight, height, waist circumference, and blood pressure (BP) were measured at mobile examination centers by trained staff according to standardized procedures.^23^ Body mass index was calculated as weight in kilograms divided by height in meters squared. Three BP measurements were assessed, and systolic BP and diastolic BP were calculated as the mean of all available measurements.

Participants were asked to provide blood samples at the mobile examination centers. The samples were stored at −20 °C and sent to central laboratories to determine lipid, plasma glucose, serum insulin, and C-reactive protein levels following standard protocols.^23^ A subset of participants were randomly selected to attend the morning session after an overnight fast; triglycerides, fasting plasma glucose (FPG), and insulin were measured for those who fasted at least 8 hours. Insulin resistance was assessed with the homeostasis model assessment score.^26^ Although there were changes in the laboratories, methods, and instruments used to measure lipid levels,^27^ all laboratories participated in the CDC Lipids Standardization Program,^28^ thus ensuring the accuracy, precision, and comparability of lipid measurements across cycles. To account for changes in laboratory methods over time, we calibrated FPG and serum insulin measurements to early cycles using the recommended backward equations.^23^

### MHO and MUO Criteria

Obesity and abdominal obesity were defined as a BMI of 30.0 or more and a waist circumference of 102 cm or more for men and 88 cm or more for women. The ethnicity-specific BMI cutoff for non-Hispanic Asian individuals was not used due to the lack of classification of this subgroup in the NHANES before 2011.^24^ Metabolic health was defined according to the harmonized definition proposed by Lavie et al^17^ and Ortega et al.^18^ Adults with obesity were classified as having MHO if they had 0 of 4 MetS components^29,30^: (1) elevated BP (systolic BP ≥130 mm Hg, diastolic BP ≥85 mm Hg, or antihypertensive medication use); (2) elevated FPG (≥100 mg/dL [to convert to millimoles per liter, multiply by 0.0555] or antidiabetic medication use); (3) reduced high-density lipoprotein cholesterol (HDL-C) (<40 mg/dL for men and <50 mg/dL for women [to convert to millimoles per liter, multiply by 0.0259]); or (4) elevated triglycerides (≥150 mg/dL [to convert to millimoles per liter, multiply by 0.0113]). Waist circumference was excluded for collinearity with BMI. Since data for cholesterol medication were available only for general use but not for treatment of elevated triglycerides or reduced HDL-C specifically, we did not utilize this information to avoid overestimation of these components, consistent with previous reports on MetS.^31^ Participants with obesity who met any of the above criteria were classified as having MUO.

### Statistical Analysis

We first evaluated trends in the prevalence of obesity, MUO, and MHO among all study participants from 1999 to 2018. Prevalence estimates were age standardized to the 2000 US Census population, using 3 age groups (20-39, 40-59, and ≥60 years) by the direct method. To calculate the number of individuals with obesity, MUO, or MHO, we next multiplied age-standardized prevalence estimates by the total noninstitutionalized adult population for each NHANES cycle.^32^ Trends in MHO proportion and individual metabolic indicators among those with obesity were then evaluated overall and by age group, sex, race and ethnicity, education level, income-to-poverty ratio, home ownership, and health insurance type. Proportion estimates were age standardized to all nonpregnant adults with obesity in the 2015-2018 NHANES cycles, using the same 3 age groups. To improve the reliability and precision of weighted estimates, 2 adjacent cycles were combined in consideration of the low prevalence of MHO. Linear trends over time were evaluated using logistic regression after regressing MHO on survey cycles (modeled as a continuous independent variable). Factors associated with metabolic health among adults with obesity were further identified with logistic regression models, adjusting for age group, sex, and race and ethnicity.

The complex survey design factors for the NHANES, including sample weights, clustering, and stratification, were accounted for as specified in the NHANES statistical analysis guideline.^24^ We used morning fasting subsample weights in all analyses to produce estimates representative of the US population. Standard errors were estimated with Taylor series linearization. Complete case analysis was applied if the missing data level for analyses was 10% or less. Several sensitivity analyses were conducted to evaluate the impact of different criteria on MHO trends. First, information on self-reported cholesterol medication use was also used to define MUO and MHO. Second, individuals with a previous diagnosis of cardiovascular disease (CVD) were regarded as having MUO, regardless of their metabolic status.^33^ Third, abdominal obesity was used as a surrogate of general obesity in the definitions of MHO and MUO. Finally, other definitions commonly used by previous studies based on MetS components,^29,30^ insulin resistance,^4^ or together with inflammation^5,6^ were used to define metabolic health (eTable 2 in Supplement 1).

All analyses were performed with SAS, version 9.4 (SAS Institute Inc). Two-sided *P* < .05 was considered statistically significant. Adjustment for multiple comparisons was not performed as in previous reports,^1,34^ and the results should be interpreted as exploratory due to the potential for type I error. Statistical analyses were conducted from November 2021 to August 2022.

## Results

This survey study included 20 430 NHANES participants with a weighted mean (SE) age of 47.1 (0.2) years; 50.8% were women and 49.2% were men. In terms of race and ethnicity, 8.2% participants self-identified as Mexican American, 10.8% as non-Hispanic Black, 68.8% as non-Hispanic White, and 12.3% as other race or ethnicity (eTable 3 in Supplement 1). Data on education, income-to-poverty ratio, home ownership, and health insurance were missing for 0.1%, 7.3%, 1.0%, and 0.6% of participants. Analyses of trends in MHO proportion and individual metabolic indicators were restricted to 7386 adults with obesity. Their weighted mean (SE) age was 48.0 (0.3) years; 53.5% were women and 46.5% were men. From the 1999-2002 to 2015-2018 cycles, the proportions of participants with some college education or more, government insurance, or higher-class obesity increased (Table 1).

**Table 1.** Weighted Characteristics of Adult Participants With Obesity in the 1999-2002 to 2015-2018 National Health and Nutrition Examination Survey Cycles

Characteristic	Percentage of adults by year (95% CI)				
	1999-2002 (n = 1073)	2003-2006 (n = 1198)	2007-2010 (n = 1725)	2011-2014 (n = 1625)	2015-2018 (n = 1765)
Age, mean (SE) [95% CI], y	46.9 (0.8) [45.3-48.6]	47.2 (0.5) [46.2-48.3]	48.2 (0.5) [47.3-49.2]	48.6 (0.6) [47.3-49.9]	48.6 (0.7) [47.2-49.9]
Age group, y					
20-39	34.0 (29.0-39.1)	33.4 (30.5-36.4)	33.0 (30.7-35.4)	32.1 (28.2-35.9)	33.6 (30.4-36.8)
40-59	43.4 (39.1-47.7)	44.0 (40.7-47.4)	40.9 (38.6-43.1)	40.5 (37.4-43.7)	37.2 (33.9-40.6)
≥60	22.6 (18.8-26.3)	22.5 (19.3-25.8)	26.1 (24.0-28.3)	27.4 (24.6-30.2)	29.1 (25.1-33.1)
Sex					
Men	45.2 (41.7-48.6)	48.1 (44.7-51.4)	47.1 (44.3-49.8)	45.1 (42.4-47.8)	46.9 (42.8-51.0)
Women	54.8 (51.4-58.3)	51.9 (48.6-55.3)	52.9 (50.2-55.7)	54.9 (52.2-57.6)	53.1 (49.0-57.2)
Race and ethnicity^a^					
Mexican American	7.5 (5.2-9.9)	8.0 (4.9-11.1)	9.2 (5.6-12.9)	11.0 (7.5-14.5)	10.8 (7.0-14.5)
Non-Hispanic Black	13.4 (9.7-17.1)	15.1 (12.0-18.2)	14.7 (11.1-18.3)	14.6 (10.6-18.5)	13.2 (9.4-17.1)
Non-Hispanic White	70.6 (66.0-75.2)	70.1 (65.0-75.1)	66.1 (59.4-72.8)	64.9 (58.6-71.2)	62.5 (56.6-68.4)
Other	8.4 (4.1-12.8)	6.8 (4.3-9.3)	10.0 (7.1-12.9)	9.5 (6.8-12.1)	13.5 (11.1-15.8)
Education level^b^					
Less than high school	21.1 (18.5-23.8)	18.7 (15.4-21.9)	22.0 (19.3-24.7)	18.4 (15.5-21.3)	13.7 (11.2-16.1)
High school or equivalent	28.7 (24.7-32.8)	28.5 (25.7-31.4)	24.4 (21.7-27.0)	22.2 (19.5-24.8)	26.6 (23.5-29.7)
Some college or more	50.1 (45.3-54.9)	52.8 (48.9-56.7)	53.7 (50.0-57.3)	59.4 (55.9-62.8)	59.7 (56.2-63.3)
Income-to-poverty ratio, %^c^					
≤100	13.2 (10.0-16.4)	10.8 (8.4-13.1)	13.9 (11.5-16.3)	17.8 (14.0-21.6)	14.1 (11.1-17.0)
101-399	54.8 (49.8-59.8)	56.0 (50.6-61.4)	53.8 (50.5-57.0)	53.7 (49.0-58.5)	51.5 (46.1-56.9)
≥400	32.0 (26.9-37.1)	33.2 (28.6-37.9)	32.4 (28.4-36.3)	28.5 (24.0-33.0)	34.5 (28.1-40.8)
Home ownership^d^					
Owned home	71.1 (65.8-76.4)	71.8 (67.3-76.4)	71.8 (68.1-75.6)	63.5 (59.5-67.6)	65.4 (60.4-70.5)
Rented home or other arrangement	28.9 (23.6-34.2)	28.2 (23.6-32.7)	28.2 (24.4-31.9)	36.5 (32.4-40.5)	34.6 (29.5-39.6)
Health insurance type^e^					
Private	71.3 (67.5-75.1)	68.1 (63.5-72.7)	66.2 (62.5-69.9)	58.6 (54.9-62.2)	62.7 (58.5-67.0)
Government	13.9 (10.8-17.0)	15.1 (12.3-17.8)	15.9 (13.4-18.4)	22.0 (18.7-25.4)	25.3 (21.7-28.8)
None	14.8 (11.8-17.8)	16.8 (13.8-19.8)	17.9 (15.0-20.8)	19.4 (16.8-22.0)	12.0 (9.2-14.8)
Weight group by obesity class (BMI range)					
Class I (30.0-34.9)	59.1 (54.2-63.9)	56.3 (53.3-59.4)	57.8 (55.7-59.9)	56.2 (52.7-59.8)	53.7 (50.4-56.9)
Class II (35.0-39.9)	26.8 (23.6-30.1)	25.6 (22.8-28.5)	26.0 (22.7-29.2)	23.8 (21.3-26.2)	26.1 (23.6-28.6)
Class III (≥40.0)	14.1 (10.6-17.6)	18.0 (15.2-20.9)	16.2 (14.0-18.4)	20.0 (17.1-22.9)	20.2 (16.9-23.5)
Abdominal obesity^f^					
Yes	96.3 (94.6-98.0)	97.4 (96.4-98.4)	96.2 (94.9-97.5)	96.2 (94.8-97.6)	97.1 (96.2-98.0)
No	3.7 (2.0-5.4)	2.6 (1.6-3.6)	3.8 (2.5-5.1)	3.8 (2.4-5.2)	2.9 (2.0-3.8)

Abbreviation: BMI, body mass index (calculated as weight in kilograms divided by height in meters squared).

^a^ Self-reported according to fixed categories and classified as Mexican American, non-Hispanic Black, non-Hispanic White, or other race and ethnicity (eg, non-Hispanic Asian or multiple races or ethnicities).

^b^ Missing for 3 participants (0%).

^c^ Missing for 659 participants (6.7%).

^d^ Missing for 78 participants (0.8%).

^e^ Missing for 45 participants (0.4%).

^f^ Missing for 183 participants (2.0%).

### Trends in MHO Prevalence Among the Population With Obesity

**Figure 1.**
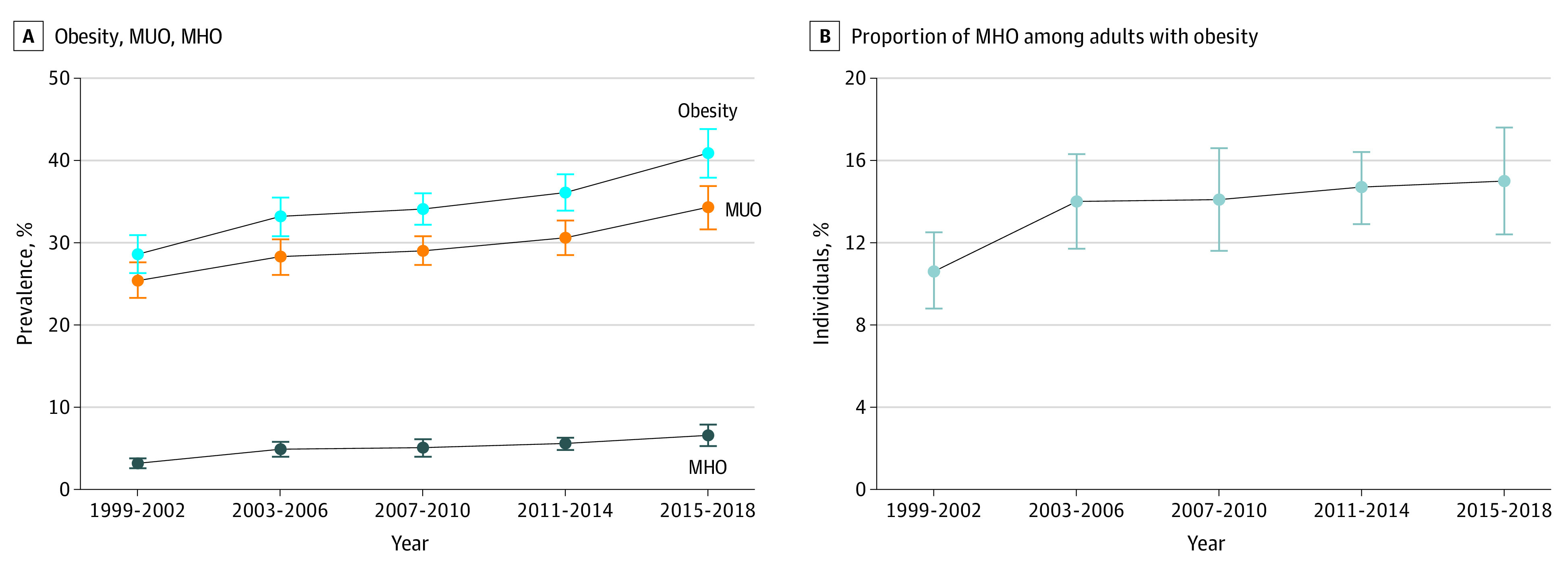
Trends in the Prevalence of Obesity, Metabolically Unhealthy Obesity (MUO), and Metabolically Healthy Obesity (MHO) Among US Adults, 1999-2018 A, Trends in the prevalence of obesity, MUO, and MHO among US adults. From 1999-2002 to 2015-2018, *P* < .001 for trend in obesity, MUO, and MHO prevalence. From 2003-2006 to 2015-2018, *P* < .001 for trend in obesity and MUO prevalence and *P =* .02 for trend in MHO prevalence. B, Trends in the proportion of MHO among US adults with obesity. From 1999-2002 to 2015-2018, *P* = .02 for trend. From 2003-2006 to 2015-2018, *P* = .51 for trend. Obesity was defined as a body mass index of 30.0 or greater (calculated as weight in kilograms divided by height in meters squared). Among participants with obesity, MUO was defined as having any component of the metabolic syndrome (waist circumference excluded) and MHO was defined as meeting none of the metabolic syndrome criteria. In A, prevalence estimates were age standardized to the 2000 US Census population, using 3 age groups (20-39, 40-59, and ≥60 years). In B, proportion estimates were age standardized to the nonpregnant adult population with obesity in the 2015-2018 National Health and Nutrition Examination Survey cycles, using the same 3 age groups. All estimates were weighted, and error bars indicate 95% CIs. Linear trends over time were evaluated using logistic regression. Specific estimates are shown in Table 2 and eTable 4 in Supplement 1.

For the whole study population, the age-standardized prevalence (95% CI) of obesity increased significantly from 28.6% (26.3%-30.9%) in the 1999-2002 cycles to 40.9% (37.9%-43.8%) in the 2015-2018 cycles (*P* < .001 for trend). The age-standardized prevalence (95% CI) of MUO also increased from 25.4% (23.3%-27.6%) in 1999-2002 to 34.3% (31.6%-36.9%) in 2015-2018 (*P* < .001 for trend). Finally, the prevalence (95% CI) of MHO increased from 3.2% (2.6%-3.8%) in 1999-2002 to 6.6% (5.3%-7.9%) in 2015-2018 (*P* < .001 for trend; Figure 1A).

Within racial and ethnic subgroups, more than 40% of Mexican American adults and non-Hispanic Black adults in the 2015-2018 cycles had MUO; however, the prevalence of MHO was low among all racial and ethnic subpopulations (eTable 4 in Supplement 1). In the 2015-2018 cycles, an estimated 81.1 million US adults (95% CI, 74.7-87.4) had MUO and 15.6 million (12.5-18.6) had MHO (eFigure 1 in Supplement 1).

Among the 7386 participants with obesity, the age-standardized proportion (95% CI) of MHO increased significantly from 10.6% (8.8%-12.5%) in the 1999-2002 cycles to 15.0% (12.4%-17.6%) in the 2015-2018 cycles (*P* = .02 for trend; Figure 1B). A substantial increase was observed among individuals aged 60 years or older, men, and non-Hispanic White adults as well as those with higher income, private insurance, or class I obesity (all *P* < .05 for trend; Table 2). However, this increase was largely attributable to an increase between the 1999-2002 and 2003-2006 cycles. When trends from the 2003-2006 to 2015-2018 cycles were evaluated, there was no significant increase in the age-standardized proportion of MHO (Table 2).

**Table 2.** Trends in the Percentage of US Adults With Metabolically Healthy Obesity (MHO) Among the Population With Obesity, 1999-2018^a^

Characteristic	Adults with MHO, % (95% CI)^b^					*P* value for trend^c^	
	1999-2002 (n = 1073)	2003-2006 (n = 1198)	2007-2010 (n = 1725)	2011-2014 (n = 1625)	2015-2018 (n = 1765)	1999-2002	2003-2006
Overall %	10.6 (8.8-12.5)	14.0 (11.7-16.3)	14.1 (11.6-16.6)	14.7 (12.9-16.4)	15.0 (12.4-17.6)	.02	.51
Age, y							
20-39	18.2 (12.9-23.4)	26.8 (20.8-32.7)	25.1 (18.9-31.4)	24.9 (20.2-29.7)	27.2 (21.7-32.8)	.09	.86
40-59	10.4 (6.7-14.1)	10.5 (6.9-14.1)	12.6 (9.4-15.8)	12.2 (8.9-15.5)	11.4 (7.5-15.3)	.56	.79
≥60	2.4 (0.5-4.4)	4.3 (2.0-6.6)	3.6 (1.5-5.7)	6.4 (3.8-8.9)	5.9 (3.0-8.9)	.03	.18
Sex							
Men	7.9 (5.2-10.6)	11.9 (8.0-15.8)	13.2 (9.7-16.7)	12.7 (9.9-15.4)	13.9 (10.2-17.5)	.04	.52
Women	12.9 (10.1-15.8)	16.1 (13.0-19.2)	14.8 (11.8-17.9)	16.2 (14.0-18.5)	16.0 (12.5-19.5)	.23	.82
Race and ethnicity^d^							
Mexican American	10.3 (7.2-13.5)	13.5 (8.6-18.5)	13.0 (9.0-17.1)	12.2 (8.6-15.8)	12.8 (9.2-16.5)	.85	.89
Non-Hispanic Black	14.7 (10.0-19.4)	19.1 (14.0-24.2)	16.7 (12.8-20.6)	16.1 (13.0-19.3)	15.5 (12.9-18.0)	.54	.13
Non-Hispanic White	7.5 (5.4-9.6)	12.5 (9.2-15.8)	13.6 (10.1-17.2)	14.5 (12.0-16.9)	15.7 (11.5-20.0)	.002	.20
Other	21.8 (12.3-31.4)	14.2 (6.5-21.9)	12.0 (7.0-17.0)	14.3 (7.6-21.0)	13.4 (10.1-16.8)	.20	.93
Education level^e^							
Less than high school	13.1 (8.4-17.9)	10.4 (5.9-14.9)	8.8 (5.3-12.3)	10.4 (6.4-14.4)	12.3 (6.3-18.2)	.79	.59
High school or equivalent	8.6 (4.3-12.8)	9.5 (5.5-13.6)	14.9 (9.9-19.9)	13.8 (10.0-17.6)	11.5 (7.4-15.6)	.15	.72
Some college or more	10.9 (7.8-14.0)	17.5 (14.5-20.6)	15.7 (12.3-19.0)	16.3 (13.9-18.8)	17.0 (13.0-21.1)	.12	.97
Income-to-poverty ratio, %^f^							
≤100	12.7 (7.6-17.8)	13.9 (6.7-21.0)	9.1 (3.4-14.7)	12.7 (8.7-16.6)	12.2 (8.9-15.5)	.94	.92
101-399	10.5 (7.5-13.6)	13.5 (10.9-16.2)	13.3 (9.8-16.9)	14.8 (11.7-18.0)	13.7 (10.3-17.1)	.22	.92
≥400	9.2 (4.6-13.8)	15.0 (10.8-19.2)	16.9 (11.7-22.1)	18.0 (13.1-22.9)	18.8 (12.8-24.8)	.03	.38
Home ownership^g^							
Owned home	11.3 (8.4-14.1)	14.8 (12.1-17.6)	13.4 (10.4-16.5)	15.7 (13.1-18.4)	15.8 (11.8-19.9)	.07	.43
Rented home or other arrangement	9.5 (5.5-13.5)	11.9 (8.4-15.4)	14.4 (10.0-18.7)	13.3 (10.0-16.6)	13.6 (10.5-16.7)	.13	.87
Health insurance type^h^							
Private	10.0 (7.9-12.1)	14.8 (11.7-18.0)	15.8 (12.9-18.7)	15.4 (12.2-18.6)	16.5 (12.8-20.3)	.01	.54
Government	9.5 (2.0-16.9)	9.3 (4.4-14.2)	6.3 (2.8-9.9)	13.0 (7.9-18.1)	13.3 (9.5-17.0)	.05	.03
None	15.9 (11.1-20.7)	13.4 (9.0-17.8)	13.5 (8.7-18.2)	17.4 (10.4-24.3)	14.5 (9.1-19.9)	.63	.44
Weight group by obesity class (BMI range)							
Class I (30.0-34.9)	12.1 (9.4-14.9)	17.5 (14.1-21.0)	16.8 (13.7-19.9)	18.8 (15.9-21.7)	18.6 (14.5-22.8)	.02	.51
Class II (35.0-39.9)	9.0 (5.1-12.8)	14.4 (9.4-19.3)	12.5 (9.3-15.6)	12.3 (8.1-16.5)	13.0 (7.6-18.4)	.48	.85
Class III (≥40.0)	7.5 (3.2-11.8)	3.8 (1.7-5.8)	7.8 (4.0-11.6)	6.4 (3.4-9.4)	8.2 (4.3-12.2)	.34	.10

Abbreviation: BMI, body mass index (calculated as weight in kilograms divided by height in meters squared).

^a^ Defined as participants with obesity who met no criteria for metabolic syndrome (waist circumference excluded). Estimates by age groups were unadjusted. Other estimates were age standardized to the nonpregnant adult population with obesity in the 2015-2018 National Health and Nutrition Examination Survey cycles, using 3 age groups (20-39, 40-59, and ≥60 years) by the direct method.

^b^ Sample sizes are unweighted. Data are presented as weighted percentages (95% CIs).

^c^ Trends over time from year ranges listed to 2015-2018 were evaluated using logistic regression.

^d^ Self-reported according to fixed categories and classified as Mexican American, non-Hispanic Black, non-Hispanic White, or other race and ethnicity (eg, non-Hispanic Asian or multiple races or ethnicities).

^e^ Missing for 3 participants (0%).

^f^ Missing for 659 participants (6.7%).

^g^ Missing for 78 participants (0.8%).

^h^ Missing for 45 participants (0.4%).

### Trends in Individual Metabolic Indicators Among the Population With Obesity

**Figure 2.**
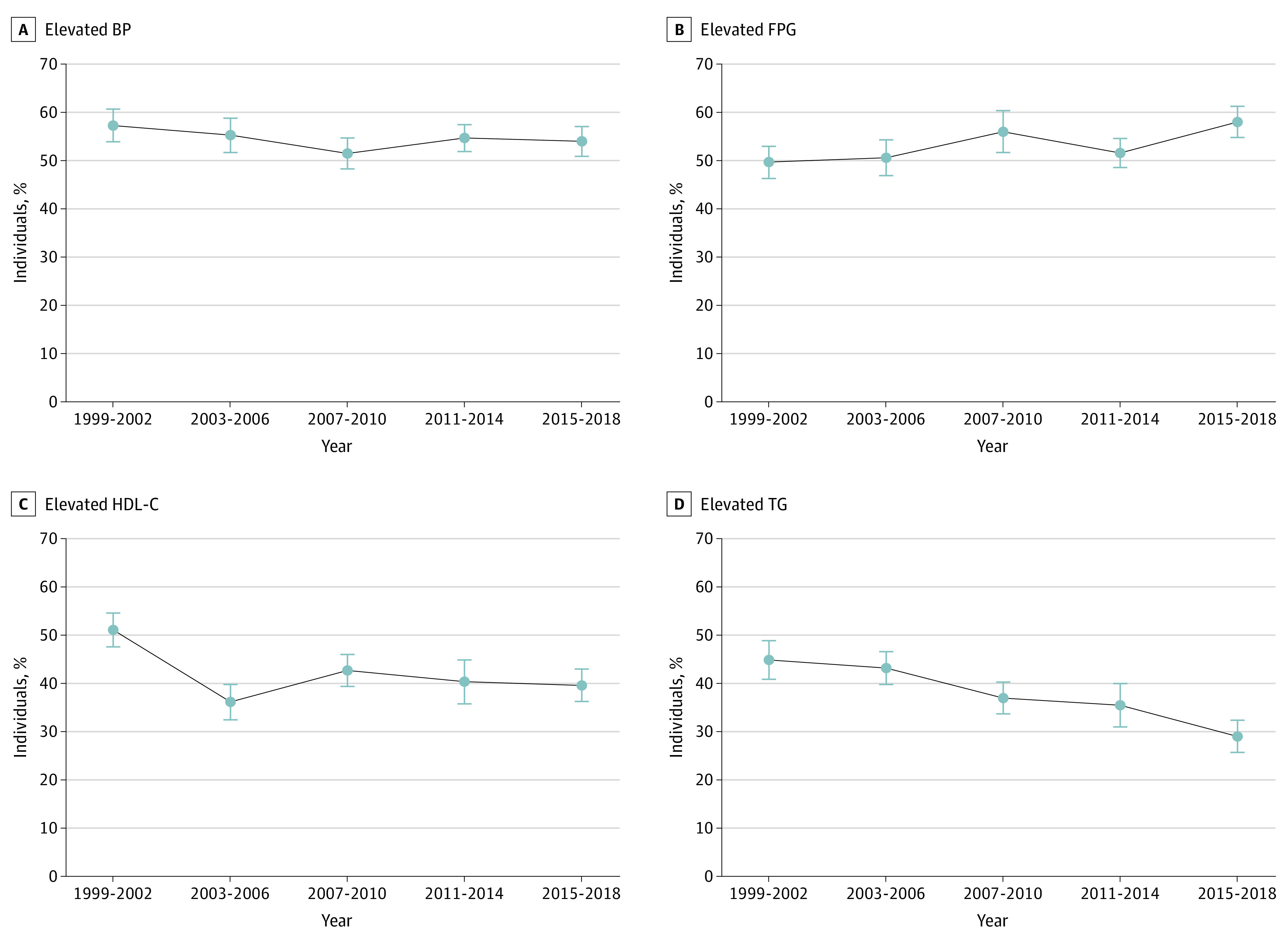
Trends in the Percentage of Individual Clinical Metabolic Parameters Among Adults With Obesity, 1999-2018 A, Elevated blood pressure (BP; systolic BP ≥130 mm Hg, diastolic BP ≥85 mm Hg, or hypertension medication use). No significant trend was observed from 1999-2002 to 2015-2018 (*P* = .28 for trend) or from 2003-2006 to 2015-2018 (*P* = .92 for trend). B, Elevated fasting plasma glucose (FPG; ≥100 mg/dL or antidiabetic medication use). A significant increasing trend was observed from 1999-2002 to 2015-2018 (*P* < .001 for trend) and from 2003-2006 to 2015-2018 (*P* = .02 for trend). C, Reduced high-density lipoprotein cholesterol (HDL-C; <40 mg/dL for men and <50 mg/dL for women). A significant decreasing trend was observed from 1999-2002 to 2015-2018 (*P* = .006 for trend) but not from 2003-2006 to 2015-2018 (*P* = .47 for trend). D, Elevated triglycerides (TG; ≥150 mg/dL). A significant decreasing trend was observed from 1999-2002 to 2015-2018 and from 2003-2006 to 2015-2018 (both *P* < .001 for trend). Percentage estimates were age standardized to the nonpregnant adult population with obesity in the 2015-2018 National Health and Nutrition Examination Survey cycles, using 3 age groups (20-39, 40-59, and ≥60 years). All estimates were weighted and the error bars indicate 95% CIs. Linear trends over time were evaluated using logistic regression.

During the past 2 decades, there was a substantial divergence in trends for clinical metabolic indicators among individuals with obesity. From the 1999-2002 to 2015-2018 cycles, significantly decreasing trends in the age-standardized percentage (95% CI) of elevated triglycerides (from 44.9% [40.9%-48.9%] to 29.0% [25.7%-32.4%]; *P* < .001 for trend) and reduced HDL-C (from 51.1% [47.6%-54.6%] to 39.6% [36.3%-43.0%]; *P* = .006 for trend) were observed. However, no significant trend in the percentage of elevated BP (from 57.3% [95% CI, 53.9%-60.7%] to 54.0% [50.9%-57.1%]; *P* = .28 for trend) was observed, whereas the percentage of elevated FPG increased significantly (from 49.7% [46.3%-53.0%] to 58.0% [54.8%-61.3%]; *P* < .001 for trend; Figure 2).

### Factors Associated With Metabolic Health Among the Population With Obesity

Among all US participants with obesity in the 1999-2018 NHANES cycles, younger adults, women, non-Hispanic Black individuals, and those with some college education or more, higher income, home ownership, or lower obesity class were generally more likely to be metabolically healthy (Table 3). Women with obesity were more likely to have reduced HDL-C but less likely to have elevated BP, FPG, and triglycerides compared with men with obesity. Non-Hispanic Black individuals with obesity were more likely to have elevated BP but less likely to have elevated triglycerides and reduced HDL-C compared with non-Hispanic White adults with obesity.

**Table 3.** Adjusted Odds Ratios for Metabolic Health Among US Adults With Obesity, 1999-2018

Characteristic	Adjusted odds ratio (95% CI)^a^				
	MHO^b^	BP not elevated^c^	FPG not elevated^d^	HDL-C not reduced^e^	Triglycerides not elevated^f^
Age, y					
20-39	6.44 (4.86-8.52)	11.37 (9.52-13.57)	6.89 (5.73-8.28)	0.48 (0.42-0.55)	1.42 (1.19-1.71)
40-59	2.54 (1.86-3.45)	2.94 (2.48-3.49)	2.25 (1.97-2.57)	0.68 (0.58-0.79)	0.97 (0.82-1.14)
≥60	1 [Reference]	1 [Reference]	1 [Reference]	1 [Reference]	1 [Reference]
Sex					
Men	1 [Reference]	1 [Reference]	1 [Reference]	1 [Reference]	1 [Reference]
Women	1.28 (1.06-1.54)	1.40 (1.22-1.61)	1.66 (1.47-1.89)	0.63 (0.54-0.73)	1.43 (1.27-1.62)
Race and ethnicity^g^					
Mexican American	0.92 (0.73-1.16)	1.38 (1.18-1.62)	0.74 (0.62-0.89)	0.99 (0.84-1.15)	0.93 (0.80-1.08)
Non-Hispanic Black	1.32 (1.10-1.59)	0.60 (0.52-0.69)	1.14 (0.98-1.32)	1.77 (1.52-2.07)	3.28 (2.78-3.86)
Non-Hispanic White	1 [Reference]	1 [Reference]	1 [Reference]	1 [Reference]	1 [Reference]
Other	1.15 (0.88-1.50)	1.17 (0.95-1.43)	0.85 (0.67-1.07)	1.16 (0.95-1.42)	1.12 (0.91-1.38)
Education level					
Less than high school	1 [Reference]	1 [Reference]	1 [Reference]	1 [Reference]	1 [Reference]
High school or equivalent	1.09 (0.81-1.47)	0.96 (0.78-1.19)	1.11 (0.92-1.34)	1.17 (0.97-1.40)	1.10 (0.92-1.31)
Some college or more	1.60 (1.21-2.10)	1.15 (0.98-1.35)	1.31 (1.11-1.54)	1.43 (1.20-1.70)	1.30 (1.11-1.52)
Income-to-poverty ratio, %					
≤100	1 [Reference]	1 [Reference]	1 [Reference]	1 [Reference]	1 [Reference]
101-399	1.21 (0.93-1.59)	1.00 (0.82-1.21)	1.19 (1.03-1.37)	1.29 (1.09-1.52)	1.16 (0.98-1.37)
≥400	1.64 (1.20-2.25)	1.06 (0.86-1.32)	1.37 (1.14-1.66)	1.64 (1.34-2.00)	1.30 (1.06-1.60)
Home ownership					
Owned home	1.22 (1.00-1.48)	1.01 (0.85-1.18)	1.08 (0.93-1.26)	1.24 (1.04-1.47)	1.08 (0.94-1.24)
Rented home or other arrangement	1 [Reference]	1 [Reference]	1 [Reference]	1 [Reference]	1 [Reference]
Health insurance type					
Private	1.08 (0.87-1.34)	0.77 (0.64-0.92)	1.06 (0.89-1.26)	1.38 (1.18-1.61)	1.14 (0.96-1.35)
Government	0.72 (0.54-0.97)	0.65 (0.52-0.81)	0.76 (0.62-0.93)	1.07 (0.90-1.27)	0.98 (0.80-1.20)
None	1 [Reference]	1 [Reference]	1 [Reference]	1 [Reference]	1 [Reference]
Weight group by obesity class (BMI range)					
Class I (30.0-34.9)	3.22 (2.39-4.33)	2.34 (1.89-2.89)	2.79 (2.30-3.40)	1.71 (1.45-2.02)	1.20 (1.00-1.42)
Class II (35.0-39.9)	1.99 (1.47-2.70)	1.78 (1.39-2.27)	1.91 (1.54-2.38)	1.20 (1.00-1.44)	1.09 (0.90-1.32)
Class III (≥40.0)	1 [Reference]	1 [Reference]	1 [Reference]	1 [Reference]	1 [Reference]

Abbreviations: BMI, body mass index (calculated as weight in kilograms divided by height in meters squared); BP, blood pressure; FPG, fasting plasma glucose; HDL-C, high-density lipoprotein cholesterol; MHO, metabolically healthy obesity.

^a^ Adjusted for age, sex, and race and ethnicity. Data were pooled from the 1999-2000 to 2017-2018 National Health and Nutrition Examination Survey cycles to provide more stable estimates.

^b^ Defined as participants with obesity who met no metabolic syndrome criteria (ie, elevated BP, elevated FPG, reduced HDL-C, or elevated triglycerides).

^c^ Defined as elevated systolic BP (≥130 mm Hg), diastolic BP (≥85 mm Hg), or antihypertensive medication use.

^d^ Defined as elevated FPG (≥100 mg/dL [to convert to millimoles per liter, multiply by 0.0555]) or antidiabetic medication use.

^e^ Defined as reduced HDL-C (<40 mg/dL for men and <50 mg/dL for women [to convert to millimoles per liter, multiply by 0.0259]).

^f^ Defined as elevated triglycerides (≥150 mg/dL [to convert to millimoles per liter, multiply by 0.0113]).

^g^ Self-reported according to fixed categories and classified as Mexican American, non-Hispanic Black, non-Hispanic White, or other race and ethnicity (eg, non-Hispanic Asian or multiple races or ethnicities).

### Sensitivity Analysis

When individuals who used cholesterol medication or had a previous CVD diagnosis were further classified as having MUO, the proportions of MHO among adults with obesity were slightly smaller because more individuals were classified into the metabolically unhealthy group (eTables 5 and 6 in Supplement 1). Trends in metabolically healthy abdominal obesity generally followed the same patterns as observed for MHO, albeit with more notable changes (eTable 7 in Supplement 1). Sample sizes for some sensitivity analyses under other MHO criteria were somewhat smaller due to missing values for certain variables. In the 2015-2018 NHANES cycles, the age-standardized prevalence (95% CI) of MHO in the total population varied from 3.5% (2.5%-4.4%) to 18.1% (16.1%-20.2%) when using other MHO definitions, and the proportion of MHO among the population with obesity varied from 8.0% (6.0%-9.9%) to 42.4% (39.6%-45.1%) (eFigure 2 and eTable 8 in Supplement 1). There were increasing trends in the prevalence of MHO under other criteria based on MetS components. However, decreasing trends were observed when insulin resistance was used to define metabolic health. Trends in age-standardized mean concentrations of all individual metabolic parameters among adults with obesity, MUO, and MHO are shown in eTable 9 in Supplement 1.

## Discussion

The results of this nationally representative survey study suggest that the age-standardized prevalence of obesity, MUO, and MHO increased significantly among US adults from 1999 to 2018. The proportion of MHO among adults with obesity and its trends varied across different criteria. When defined as the absence of all MetS components, the proportion of MHO increased significantly from 10.6% in 1999-2002 to 15.0% in 2015-2018. However, this increase was largely due to an increase between 1999-2002 and 2003-2006, and disparities existed among sociodemographic subgroups. Our results suggest that the overall increase in MHO was driven primarily by the decrease in dyslipidemia (ie, elevated triglycerides and reduced HDL-C) among adults with obesity; however, elevated BP remained stable and elevated FPG increased during the past 2 decades.

Different MHO criteria used in previous studies have led to large discrepancies in estimates of MHO prevalence, which precludes direct comparisons among studies. Previous reviews reported that the proportion of MHO among the population with obesity ranged between 6% and 40%, depending on the criteria used.^7,8,33^ From a clinical and public health point of view, we used strict criteria based on BMI and MetS components to define MHO in our main analyses.^17,18^ Our estimates of MHO prevalence among US adults (range, 3.2%-6.6% across years) and MHO proportion among the population with obesity (range, 10.6%-15.0%) were consistent with previous reports using the same criteria.^14,15^ One study based on 2009-2016 NHANES data reported a smaller proportion of MHO (6.8%), mainly because the investigators used 120/80 mm Hg as the cut point for elevated BP.^13^ Unsurprisingly, our estimates were lower than those in studies with looser MHO criteria^4,5,6^; however, research has shown that most studies have overestimated the prevalence of MHO.^3,33^ Large heterogeneity in MHO prevalence estimates using different definitions underscores the need to establish a standardized definition of this obesity phenotype.

We have reported, to our knowledge, the most recent and comprehensive national trend estimates of MHO. The observation that MHO proportions increased from 1999 to 2018 should be treated with caution, as trends between the 2003-2006 and 2015-2018 cycles were relatively stable. These results may be better interpreted when combined with trends in individual metabolic indicators. For example, the overall increase in MHO may be driven primarily by the decrease in dyslipidemia among the population with obesity, which has also been observed for the population overall.^27,35^ A plausible explanation may include increased awareness, diagnosis, and treatment of dyslipidemia as well as decreased smoking, removal of *trans*-fatty acids from foods, and improved diet quality.^27,36,37^ In contrast, the plateau in the proportion of MHO from 2003-2006 to 2015-2018 may result from a combination of leveling off of reduced HDL-C, no significant change in elevated BP, and the significant increase in elevated FPG over the same period. Previous studies examining trends in cardiovascular health metrics among US adults with obesity have reported the following: decreases for untreated hypertension and untreated dyslipidemia between 1999 and 2010^38^; nonsignificant changes in elevated BP and improvements in mean HDL-C, but deteriorations in mean hemoglobin A_1c_ between 1988 and 2014^37^; and increases in the proportion of individuals without prior cardiovascular events or cardiometabolic diseases between 1999 and 2016.^39^ Although different time periods may contribute to variations in trend estimates, our results were generally consistent with these findings. Given the complex interplay between obesity and glucose control, greater attention should be paid to the increase in elevated FPG among adults with obesity.^40^ Beyond conventional risk factors, our study further complemented a recent study on trends in metabolic phenotypes defined by MetS components by incorporating insulin resistance and chronic inflammation to capture a wider breadth of metabolic abnormalities.^16^ It is noteworthy that the use of insulin resistance to define poor metabolic health mitigated or even reversed the overall increasing trends in MHO, which may be linked to an increase in sedentary time, waist circumference, and nonalcoholic fatty liver disease.^41,42,43^ Although reasons for these trends may be complex and warrant further investigation, these results highlight the importance of reinforcing glucose management and reducing insulin resistance among adults with obesity.

The overall increase in the proportion of MHO should also be treated in the context of existing disparities in subpopulations. Among racial and ethnic subgroups, we observed a significant increase in the proportion of MHO only in non-Hispanic White adults, which may be attributed in part to higher income, wider insurance coverage, more accessible health services, sociocultural differences, and other social determinants.^44,45,46^ Previous studies have suggested that higher-income groups tend to have improved diet quality,^36^ increased adherence to physical activity guidelines,^43^ and decreased smoking prevalence,^25^ which may contribute to favorable trends in the proportion of MHO. In contrast, adults with lower levels of education or lower income were more likely to be metabolically unhealthy; this is important to note given their already higher prevalence of obesity and lack of weight self-awareness.^47,48^ The disproportionate prevalence of and trends in metabolic alterations could aggravate obesity disparities, as these are all CVD risk factors; thus, these findings underscore the urgency for more accessible strategies to reach racial and ethnic minority individuals and those residing in low-income communities.

Although there is no consensus on the protective effect of MHO compared with metabolically healthy normal weight,^10,49,50^ accumulating evidence suggests that individuals with MHO have a better CVD prognosis than their MUO counterparts.^12,17,33^ Previous studies suggest that mechanisms including visceral and ectopic fat accumulation, adipose dysfunction, insulin resistance, inflammatory dysregulation, and gut microbiota may play a part.^33,51^ However, MHO has been considered a transitory state for most individuals with obesity, and those whose status converts to MUO would have higher risk.^9,22^ Therefore, detailed and repeated metabolic phenotyping among adults with obesity should be taken into consideration in clinical risk assessment to improve the inherent shortcomings of BMI assessment and to help those with MHO maintain their status.^8^ It should also be emphasized that although the proportion of MHO increased in this study, the absolute number of adults with MUO has increased dramatically in the past 2 decades, suggesting that MUO is still a major health concern. Effective strategies to address the double burden of obesity and metabolic disorders and to curb the increase in MUO are important.

### Limitations

This survey study has several limitations. First, there is no universally accepted definition of MHO; thus, we provided estimates under several commonly used criteria. Second, misclassification of MHO was possible because metabolic parameters such as glycemic levels and lipids were measured only once, particularly considering the transient nature of MHO.^22^ Third, we did not evaluate physical activity, cardiovascular fitness, and body fat distribution due to inconsistent or lacking assessments across survey cycles, which might be important in understanding the metabolic health status of individuals with obesity.^9,49^ Fourth, the response rate declined across surveys. Finally, although 2 adjacent NHANES cycles were combined, there was a possibility of insufficient power to detect variabilities over time, particularly in some subgroups with limited sample size.

## Conclusions

In this cross-sectional study of US adults, we observed a low prevalence of MHO and a large, increasing burden of MUO. Although the proportion of MHO among adults with obesity increased during the past 2 decades, disparities among sociodemographic subpopulations were observed. These results highlight the need for effective strategies to optimize metabolic status and prevent obesity-related complications among people with obesity, especially among vulnerable subpopulations. Priority should be placed on reinforcing glucose management and reducing insulin resistance among individuals with obesity.

## Notes

Supplement 1. **eTable 1.** Sample Selection for the Main Analysis From the Fasting Subsample of Nonpregnant Adults Aged 20 Years or Older, 1999-2018 National Health and Nutrition Examination Survey **eTable 2.** Criteria of Metabolic Health Among Adults in the Sensitivity Analysis **eTable 3.** Weighted Characteristics of All Adult Participants in the 1999-2002 to 2015-2018 National Health and Nutrition Examination Survey Cycles **eTable 4.** Trends in the Prevalence of Obesity, Metabolically Unhealthy Obesity (MUO), and Metabolically Healthy Obesity (MHO) Among US Adults, Overall and by Sex and Race and Ethnicity **eTable 5.** Trends in the Percentage of US Adults Without Any Metabolic Abnormalities or Self-reported Use of Medication for Hypertension, Hyperglycemia, or High Cholesterol, 1999-2018 **eTable 6.** Trends in the Percentage of US Adults Without Any Metabolic Abnormalities or Previous Diagnosis of Cardiovascular Disease Among the Population With Obesity, 1999-2018 **eTable 7.** Trends in the Percentage of US Adults With Metabolically Healthy Abdominal Obesity Among the Population With Abdominal Obesity, 1999-2018 **eTable 8.** Trends in the Prevalence and Proportion of Metabolically Healthy Obesity (MHO) Among US Adults With Obesity by Different Criteria, 1999-2018 **eTable 9.** Trends in Age-Adjusted Mean Concentrations of Individual Metabolic Parameters by Metabolic Health Status Among US Adults With Obesity, 1999-2018 **eFigure 1.** Number of US Adults With Obesity, Metabolically Unhealthy Obesity (MUO), or Metabolically Healthy Obesity (MHO), 1999-2018 **eFigure 2.** Trends in the Prevalence of Metabolically Healthy Obesity (MHO) Among US Adults With Obesity by Different Criteria, 1999-2018
eReferences

Supplement 2.
Data Sharing Statement
