# Supplementary material for: Trends in the Prevalence of Metabolically Healthy Obesity Among US Adults, 1999-2018
Source: JAMA Netw Open. 2023 Mar 9;6(3):e232145. doi: 10.1001/jamanetworkopen.2023.2145 (PMC9999245; doi:10.1001/jamanetworkopen.2023.2145)
Supplement: Supplement 1. — eTable 1. Sample Selection for the Main Analysis From the Fasting Subsample of Nonpregnant Adults Aged 20 Years or Older, 1999-2018 National Health and Nutrition Examination Survey eTable 2. Criteria of Metabolic Health Among Adults in the Sensitivity Analysis eTable 3. Weighted Characteristics of All Adult Participants in the 1999-2002 to 2015-2018 National Health and Nutrition Examination Survey Cycles eTable 4. Trends in the Prevalence of Obesity, Metabolically Unhealthy Obesity (MUO), and Metabolically Healthy Obesity (MHO) Among US Adults, Overall and by Sex and Race and Ethnicity eTable 5. Trends in the Percentage of US Adults Without Any Metabolic Abnormalities or Self-reported Use of Medication for Hypertension, Hyperglycemia, or High Cholesterol, 1999-2018 eTable 6. Trends in the Percentage of US Adults Without Any Metabolic Abnormalities or Previous Diagnosis of Cardiovascular Disease Among the Population With Obesity, 1999-2018 eTable 7. Trends in the Percentage of US Adults With Metabolically Healthy Abdominal Obesity Among the Population With Abdominal Obesity, 1999-2018 eTable 8. Trends in the Prevalence and Proportion of Metabolically Healthy Obesity (MHO) Among US Adults With Obesity by Different Criteria, 1999-2018 eTable 9. Trends in Age-Adjusted Mean Concentrations of Individual Metabolic Parameters by Metabolic Health Status Among US Adults With Obesity, 1999-2018 eFigure 1. Number of US Adults With Obesity, Metabolically Unhealthy Obesity (MUO), or Metabolically Healthy Obesity (MHO), 1999-2018 eFigure 2. Trends in the Prevalence of Metabolically Healthy Obesity (MHO) Among US Adults With Obesity by Different Criteria, 1999-2018 eReferences [file jamanetwopen-e232145-s001.pdf]

Supplementary Online Content

Wang JS, Xia PF, Ma MN, et al. Trends in the prevalence of metabolically healthy obesity among US adults, 1999-2018. *JAMA Netw Open*. 2023;6(3):e232145. doi:10.1001/jamanetworkopen.2023.2145

eTable 1. Sample Selection for the Main Analysis From the Fasting Subsample of Nonpregnant Adults Aged 20 Years or Older, 1999-2018 National Health and Nutrition Examination Survey
eTable 2. Criteria of Metabolic Health Among Adults in the Sensitivity Analysis
eTable 3. Weighted Characteristics of All Adult Participants in the 1999-2002 to 2015-2018 National Health and Nutrition Examination Survey Cycles
eTable 4. Trends in the Prevalence of Obesity, Metabolically Unhealthy Obesity (MUO), and Metabolically Healthy Obesity (MHO) Among US Adults, Overall and by Sex and Race and Ethnicity
eTable 5. Trends in the Percentage of US Adults Without Any Metabolic Abnormalities or Self-reported Use of Medication for Hypertension, Hyperglycemia, or High Cholesterol, 1999-2018
eTable 6. Trends in the Percentage of US Adults Without Any Metabolic Abnormalities or Previous Diagnosis of Cardiovascular Disease Among the Population With Obesity, 1999-2018
eTable 7. Trends in the Percentage of US Adults With Metabolically Healthy Abdominal Obesity Among the Population With Abdominal Obesity, 1999-2018
eTable 8. Trends in the Prevalence and Proportion of Metabolically Healthy Obesity (MHO) Among US Adults With Obesity by Different Criteria, 1999-2018
eTable 9. Trends in Age-Adjusted Mean Concentrations of Individual Metabolic Parameters by Metabolic Health Status Among US Adults With Obesity, 1999-2018
eFigure 1. Number of US Adults With Obesity, Metabolically Unhealthy Obesity (MUO), or Metabolically Healthy Obesity (MHO), 1999-2018
eFigure 2. Trends in the Prevalence of Metabolically Healthy Obesity (MHO) Among US Adults With Obesity by Different Criteria, 1999-2018
eReferences

This supplementary material has been provided by the authors to give readers additional information about their work.

© 2023 Wang JS et al. *JAMA Network Open*.

eTable 1. Sample Selection for the Main Analysis From the Fasting Subsample of Nonpregnant Adults Aged 20 Years or Older, 1999-2018 National Health and Nutrition Examination Survey

Survey wave	aged ≥20 years, nonpregnant	Fasting subsample[a]	Not fulfilling the fasting criteria[a]	Missing values in: BMI	BP	FPG	HDL-C	Triglycerides	Final analytic sample: N = (2) – Sum ([3]:[8])	Adults with obesity
	(1)	(2)	(3)	(4)	(5)	(6)	(7)	(8)		
1999-2000	4597	2060	314	12	31	5	25	1	1672	525
2001-2002	5094	2360	275	83	52	5	31	0	1914	548
2003-2004	4808	2139	252	28	96	10	8	2	1743	577
2005-2006	4643	2119	283	34	58	0	12	7	1725	621
2007-2008	5878	2750	413	40	70	0	8	2	2217	796
2009-2010	6150	2894	299	23	101	0	26	2	2443	929
2011-2012	5503	2601	307	26	77	0	26	1	2164	788
2013-2014	5704	2646	276	22	66	0	21	1	2260	837
2015-2016	5649	2562	316	17	40	0	14	2	2173	882
2017-2018	5514	2520	241	37	88	0	35	0	2119	883

Abbreviation: BMI, body mass index (calculated as weight in kilograms divided by height in meters squared); BP, blood pressure; FPG, fasting plasma glucose; HDL-C, high-density lipoprotein cholesterol.

[a] A subset of participants were randomly selected to attend the morning session after an overnight fast in the National Health and Nutrition Examination Survey. Those examined in an afternoon or evening session were not in the fasting subsample with missing values for the fasting sample weight. Those examined in a morning session, who had fasted 8-23 hours before their examination with valid plasma fasting glucose readings have non-zero fasting sample weights. All other sampled participants examined in a morning session have zero values for the fasting sample weight.[1] We excluded those with zero fasting weight to only include those who fulfilled the fasting criteria with valid plasma fasting glucose readings.

© 2023 Wang JS et al. *JAMA Network Open*.

eTable 2. Criteria of Metabolic Health Among Adults in the Sensitivity Analysis

Variable	Based on MetS components		Based on IR		Based on MetS components, IR, and inflammation	
	Criteria 1	Criteria 2	Criteria 3	Criteria 4 [a]	Criteria 5 [b]	Criteria 6 [c]
Metabolic indicators						
BP (mm Hg)	≥130/85 [d]	≥130/85 [d]	-	-	-	≥130/85 [d]
FPG (mmol/L)	≥5.6 [e]	≥5.6 [e]	-	-	-	≥5.6 [e]
HDL-C (mmol/L)	<1.0(men)<br><1.3(women)	<1.0(men)<br><1.3(women)	-	-	≥1.0(men)<br>≥1.3(women)	<1.0(men)<br><1.3(women)
Triglycerides (mmol/L)	≥1.7	≥1.7	-	-	≤1.7	≥1.7
waist circumference (cm)	≥102(men)<br>≥88(women)	-	-	-	-	-
HOMA-IR	-	-	≥2.5	$\geq P_{75th}$	≤2.7	$>P_{90th}$
CRP (mg/L)	-	-	-	-	≤3.0	$>P_{90th}$
LDL-C (mmol/L)	-	-	-	-	≤2.6	-
Criteria for MH	<3 of the above	<2 of the above	None	None	≥4 of the above	<2 of the above
Reference	[2-4]	[5]	[5]	[4]	[6]	[7]

Abbreviation: MetS, metabolic syndrome; IR, insulin resistance, evaluated based on the homeostasis model assessment (HOMA),[8] following the formula of fasting insulin (μU/mL) × fasting glucose (mg/dL)/405; BP, blood pressure; FPG, fasting plasma glucose; HDL-C, high-density lipoprotein cholesterol; CRP, C-reactive protein; LDL-C, low-density lipoprotein cholesterol; MH, metabolic health; MHO, metabolically healthy obesity; MUO, metabolically unhealthy obesity.

[a] The original study was conducted in participants without diabetes. To avoid overestimating the prevalence of MHO, diabetic participants (defined as FPG ≥7.0 mmol/L, or self-reported diagnosis of diabetes) were classified into MUO in our estimates. The 75th percentile of HOMA-IR value relates to all participants without diabetes.

[b] The original study excluded diabetics or hypertensive individuals. To avoid overestimating the prevalence of MHO, diabetics (defined as FPG ≥7.0 mmol/L, or self-reported diagnosis of diabetes) and hypertensive (defined as BP ≥140/90 mmHg, or currently antihypertensive medication use) participants were classified into MUO in our estimates.

© 2023 Wang JS et al. *JAMA Network Open*.

And the original study included only women with obesity and set the cutoff of HDL-C only for women. Because we included both men and women, different cutoffs of HDL-C for men and women were used as other studies.

[c] The 90th percentile of HOMA-IR, and CRP value relates to all participants, including those being normal weight, overweight or obesity.

[d] Antihypertensive medication use is an alternate indicator.

[e] Antidiabetic medication use is an alternate indicator.

© 2023 Wang JS et al. *JAMA Network Open*.

eTable 3. Weighted Characteristics of All Adult Participants in the 1999-2002 to 2015-2018 National Health and Nutrition Examination Survey Cycles

Characteristic	Mean (SE) or percentage (95% CI) of adults by year				
	1999-2002 (N = 3586)	2003-2006 (N = 3468)	2007-2010 (N = 4660)	2011-2014 (N = 4424)	2015-2018 (N = 4292)
Age, mean (SE), y	45.9 (0.5)	46.5 (0.6)	47.1 (0.4)	47.6 (0.5)	48.2 (0.5)
Age, y					
20-39	39.9 (37.0-42.7)	38.0 (35.5-40.4)	36.5 (33.8-39.2)	35.8 (32.9-38.7)	35.3 (33.4-37.3)
40-59	38.5 (36.5-40.6)	39.3 (37.3-41.3)	39.1 (37.0-41.2)	37.7 (35.2-40.2)	35.6 (33.1-38.1)
≥60	21.6 (19.5-23.8)	22.7 (19.8-25.7)	24.4 (22.8-25.9)	26.5 (24.5-28.6)	29.0 (26.2-31.9)
Sex					
Men	49.6 (48.1-51.1)	49.5 (48.0-51.0)	49.0 (47.4-50.6)	49.0 (47.2-50.7)	48.9 (47.3-50.5)
Women	50.4 (48.9-51.9)	50.5 (49.0-52.0)	51.0 (49.4-52.6)	51.0 (49.3-52.8)	51.1 (49.5-52.7)
Race and ethnicity [a]					
Mexican American	6.9 (5.1-8.8)	7.8 (5.3-10.2)	8.3 (5.9-10.8)	8.5 (6.0-10.9)	9.0 (6.1-12.0)
Non-Hispanic Black	10.1 (7.4-12.7)	10.8 (8.3-13.3)	10.7 (8.5-12.9)	11.3 (8.6-13.9)	11.1 (8.1-14.1)
Non-Hispanic White	72.9 (68.7-77.2)	72.6 (68.0-77.3)	69.6 (64.9-74.3)	66.8 (61.6-71.9)	63.2 (58.2-68.2)
Other	10.1 (6.2-13.9)	8.8 (6.5-11.1)	11.3 (8.7-13.9)	13.5 (11.1-16.0)	16.7 (14.2-19.2)
Education level [b]					
Less than high school	20.9 (19.1-22.6)	17.3 (14.9-19.7)	18.8 (16.9-20.7)	16.7 (13.9-19.6)	13.2 (10.9-15.5)
High School or Equivalent	26.3 (23.4-29.1)	26.4 (24.6-28.2)	23.6 (21.3-26.0)	19.9 (17.3-22.5)	25.0 (22.6-27.4)
Some college or more	52.8 (49.4-56.3)	56.3 (53.2-59.3)	57.5 (54.0-61.1)	63.4 (59.3-67.5)	61.8 (58.0-65.6)
Income-to-poverty ratio, % [c]					

© 2023 Wang JS et al. *JAMA Network Open*.

Characteristic	Mean (SE) or percentage (95% CI) of adults by year				
	**1999-2002 (N = 3586)**	**2003-2006 (N = 3468)**	**2007-2010 (N = 4660)**	**2011-2014 (N = 4424)**	**2015-2018 (N = 4292)**
≤100	12.1 (9.9-14.3)	10.3 (8.6-12.0)	13.3 (11.6-15.1)	16.2 (13.2-19.2)	13.1 (11.3-14.9)
101-399	50.0 (46.5-53.4)	53.4 (50.3-56.5)	50.2 (47.0-53.3)	49.6 (45.8-53.4)	50.2 (47.0-53.5)
≥400	38.0 (33.6-42.3)	36.2 (33.1-39.4)	36.5 (33.4-39.6)	34.2 (29.1-39.2)	36.7 (32.9-40.5)
Home ownership [d]					
Owned home	70.2 (66.1-74.3)	69.9 (65.3-74.6)	69.9 (67.3-72.5)	65.4 (61.7-69.0)	67.4 (64.0-70.8)
Rented home or other arrangement	29.8 (25.7-33.9)	30.1 (25.4-34.7)	30.1 (27.5-32.7)	34.6 (31.0-38.3)	32.6 (29.2-36.0)
Health insurance type [e]					
Private	71.2 (68.8-73.6)	66.1 (62.6-69.7)	67.0 (65.0-69.1)	61.5 (57.8-65.2)	62.4 (58.9-65.8)
Government	12.1 (10.8-13.5)	14.7 (12.8-16.6)	13.3 (11.6-14.9)	19.4 (16.9-21.9)	23.4 (20.9-25.9)
None	16.7 (14.4-19.0)	19.2 (16.3-22.1)	19.7 (18.2-21.2)	19.1 (17.0-21.2)	14.2 (11.5-16.9)
Weight group (BMI range, kg/m^2)					
Underweight (<18.5)	1.9 (1.2-2.5)	1.6 (1.1-2.2)	1.7 (1.1-2.2)	1.5 (1.2-1.9)	1.5 (1.0-2.0)
Normal weight (18.5-24.9)	34.7 (32.5-36.9)	31.7 (29.7-33.8)	30.4 (28.4-32.3)	29.0 (26.6-31.3)	25.9 (23.5-28.3)
Overweight (24.9-29.9)	34.8 (32.4-37.1)	33.3 (31.0-35.6)	33.7 (31.9-35.5)	33.2 (31.4-34.9)	31.7 (29.8-33.5)
Obesity (≥30.0)	28.6 (26.3-31.0)	33.3 (31.0-35.7)	34.3 (32.3-36.2)	36.3 (34.1-38.6)	40.9 (38.1-43.8)
Abdominal obesity [f]					
Yes	46.5 (44.0-48.9)	53.7 (51.0-56.4)	53.7 (51.3-56.0)	56.3 (53.7-58.8)	59.9 (56.6-63.2)
No	53.5 (51.1-56.0)	46.3 (43.6-49.0)	46.3 (44.0-48.7)	43.7 (41.2-46.3)	40.1 (36.8-43.4)

Abbreviation: SE, standard error; CI, Confidence Interval; BMI, body mass index (calculated as weight in kilograms divided by height in meters squared).

© 2023 Wang JS et al. *JAMA Network Open*.

[a] Self-reported according to fixed categories and classified as Mexican American, non-Hispanic Black, non-Hispanic White or other race and ethnicity (eg, non-Hispanic Asian or multiple).

[b] Missing for 18 participants (0.1%).

[c] Missing for 1868 participants (7.3%).

[d] Missing for 264 participants (1.0%).

[e] Missing for 147 participants (0.6%).

[f] Missing for 395 participants (1.6%).

© 2023 Wang JS et al. *JAMA Network Open*.

eTable 4. Trends in the Prevalence of Obesity, Metabolically Unhealthy Obesity (MUO), and Metabolically Healthy Obesity (MHO) Among US Adults, Overall and by Sex and Race and Ethnicity[a]

	Percentage (95% CI) of adults by year					*P* for trend[b]	
	1999-2002 (N = 3586)	2003-2006 (N = 3468)	2007-2010 (N = 4660)	2011-2014 (N = 4424)	2015-2018 (N = 4292)	1999-2002	2003-2006
**Overall**							
Prevalence of Obesity	28.6 (26.3-30.9)	33.2 (30.8-35.5)	34.1 (32.2-36.0)	36.1 (33.9-38.3)	40.9 (37.9-43.8)	<.001	<.001
Prevalence of MUO	25.4 (23.3-27.6)	28.3 (26.1-30.4)	29.0 (27.3-30.8)	30.6 (28.5-32.7)	34.3 (31.6-36.9)	<.001	<.001
Prevalence of MHO	3.2 (2.6-3.8)	4.9 (4.0-5.8)	5.1 (4.0-6.1)	5.6 (4.8-6.3)	6.6 (5.3-7.9)	<.001	.02
**Men**							
Prevalence of Obesity	26.3 (23.6-28.9)	32.3 (29.2-35.3)	32.9 (29.8-35.9)	33.4 (30.7-36.0)	39.2 (34.3-44.0)	<.001	.01
Prevalence of MUO	24.2 (21.5-26.9)	28.2 (25.1-31.3)	28.3 (25.5-31.2)	28.9 (26.6-31.1)	33.4 (29.6-37.3)	<.001	.03
Prevalence of MHO	2.1 (1.4-2.8)	4.1 (2.7-5.4)	4.5 (3.2-5.9)	4.5 (3.4-5.7)	5.7 (3.8-7.6)	<.001	.15
**Women**							
Prevalence of Obesity	31.1 (28.0-34.2)	34.1 (31.2-36.9)	35.4 (33.4-37.4)	38.8 (35.9-41.8)	42.5 (38.9-46.1)	<.001	<.001
Prevalence of MUO	26.8 (24.1-29.6)	28.3 (25.8-30.7)	29.8 (27.7-31.8)	32.3 (29.6-35.1)	35.1 (31.4-38.7)	<.001	<.001
Prevalence of MHO	4.3 (3.2-5.4)	5.8 (4.4-7.2)	5.6 (4.3-6.9)	6.5 (5.5-7.5)	7.4 (5.7-9.2)	.001	.07
**Mexican American**							
Prevalence of Obesity	32.2 (28.7-35.7)	35.3 (31.7-38.9)	39.0 (34.5-43.5)	47.7 (42.1-53.2)	48.9 (44.3-53.5)	<.001	<.001
Prevalence of MUO	28.6 (26.0-31.2)	30.3 (26.3-34.3)	33.8 (29.4-38.1)	41.3 (36.2-46.4)	42.2 (38.6-45.7)	<.001	<.001
Prevalence of MHO	3.6 (2.1-5.0)	5.0 (3.3-6.8)	5.2 (3.5-7.0)	6.4 (4.5-8.3)	6.7 (4.3-9.2)	.02	.14
**Non-Hispanic Black**							
Prevalence of Obesity	38.9 (34.7-43.1)	46.9 (42.8-51.1)	46.9 (42.9-50.9)	47.0 (43.2-50.9)	48.9 (45.3-52.6)	.001	.42

© 2023 Wang JS et al. *JAMA Network Open*.

	Percentage (95% CI) of adults by year					*P* for trend [b]	
	1999-2002 (N = 3586)	2003-2006 (N = 3468)	2007-2010 (N = 4660)	2011-2014 (N = 4424)	2015-2018 (N = 4292)	1999-2002	2003-2006
Prevalence of MUO	32.7 (28.3-37.2)	36.9 (33.7-40.0)	38.0 (34.0-42.1)	38.7 (35.5-41.8)	41.1 (38.1-44.1)	<.001	.04
Prevalence of MHO	6.2 (4.1-8.2)	10.1 (6.9-13.3)	8.9 (6.6-11.2)	8.4 (6.4-10.4)	7.8 (6.2-9.5)	.72	.18
**Non-Hispanic White**							
Prevalence of Obesity	27.4 (24.3-30.5)	31.6 (28.8-34.5)	32.0 (29.2-34.7)	34.7 (32.0-37.4)	40.1 (35.8-44.4)	<.001	<.001
Prevalence of MUO	25.2 (22.3-28.2)	27.6 (24.8-30.3)	27.4 (25.0-29.9)	29.6 (27.0-32.1)	33.4 (29.4-37.3)	<.001	.005
Prevalence of MHO	2.1 (1.5-2.8)	4.1 (2.9-5.2)	4.5 (3.2-5.8)	5.1 (4.2-6.0)	6.7 (4.7-8.8)	<.001	.01
**Other race/ethnicity [c]**							
Prevalence of Obesity	23.6 (17.0-30.1)	25.6 (18.2-33.1)	30.4 (25.4-35.4)	25.8 (20.6-30.9)	33.0 (29.2-36.8)	.03	.12
Prevalence of MUO	18.0 (13.6-22.4)	21.5 (14.6-28.5)	26.4 (22.1-30.8)	21.9 (17.4-26.4)	28.0 (24.5-31.4)	.004	.18
Prevalence of MHO	5.6 (2.4-8.7)	4.1 (1.8-6.4)	4.0 (2.2-5.7)	3.8 (1.7-5.9)	5.1 (3.8-6.4)	.87	.38

Abbreviation: CI, Confidence Interval.

[a] Obesity was defined as a body mass index ≥ 30 kg/m^2. MUO was defined as having any component of the metabolic syndrome (waist circumference excluded) among participants with obesity. MHO was defined as meeting none of the metabolic syndrome criteria among participants with obesity. The prevalence estimates were age-standardized to the 2000 U.S. Census population, using 3 age groups (20-39, 40-59, and ≥60 years) by the direct method.

[b] Trends over time from year ranges listed to 2015-2018 were evaluated using logistic regression.

[c] Race/ethnicity was self-reported according to fixed categories; The "Other" category included race/ethnicity other that Mexican American, non-Hispanic Black, and non-Hispanic White, including non-Hispanic Asian, multiracial.

© 2023 Wang JS et al. *JAMA Network Open*.

eTable 5. Trends in the Percentage of US Adults Without Any Metabolic Abnormalities or Self-reported Use of Medication for Hypertension, Hyperglycemia, or High Cholesterol, 1999-2018[a]

Characteristic	Adults with MHO without self-reported use of medication for hypertension, hyperglycemia or high cholesterol, % (95% CI)[b]					*P* for trend[c]	
	**1999-2002 (n = 1073)**	**2003-2006 (n = 1198)**	**2007-2010 (n = 1725)**	**2011-2014 (n = 1625)**	**2015-2018 (n = 1765)**	**1999-2002**	**2003-2006**
Overall percentage	10.4 (8.5-12.2)	13.7 (11.4-15.9)	13.6 (11.0-16.1)	13.8 (11.9-15.8)	14.8 (12.2-17.4)	.02	.47
Age, y							
20-39	18.2 (12.9-23.4)	26.6 (20.7-32.5)	25.1 (18.9-31.4)	24.9 (20.2-29.7)	27.2 (21.7-32.8)	.08	.83
40-59	9.8 (6.1-13.5)	10.1 (6.6-13.7)	11.5 (8.3-14.8)	11.4 (8.2-14.6)	11.3 (7.4-15.3)	.47	.68
≥60	2.4 (0.5-4.4)	3.8 (1.7-5.9)	3.2 (1.1-5.3)	4.5 (2.3-6.7)	5.4 (2.4-8.3)	.09	.24
Sex							
Men	7.9 (5.2-10.6)	11.7 (7.8-15.6)	13.0 (9.4-16.5)	12.7 (9.9-15.4)	13.7 (10.1-17.3)	.04	.48
Women	12.5 (9.6-15.3)	15.7 (12.6-18.7)	14.1 (11.1-17.0)	14.7 (12.2-17.3)	15.8 (12.2-19.4)	.26	.80
Race and ethnicity[d]							
Mexican American	10.3 (7.2-13.5)	12.6 (7.5-17.8)	12.1 (8.5-15.6)	12.2 (8.6-15.8)	12.8 (9.2-16.5)	.70	.86
Non-Hispanic Black	14.7 (10.0-19.4)	18.7 (13.9-23.5)	16.7 (12.8-20.6)	15.9 (12.9-18.8)	14.9 (12.4-17.4)	.47	.10
Non-Hispanic White	7.1 (5.0-9.3)	12.2 (8.8-15.7)	12.9 (9.3-16.6)	13.4 (10.5-16.2)	15.6 (11.3-19.9)	.002	.20
Other	21.8 (12.3-31.4)	14.2 (6.5-21.9)	11.8 (6.8-16.9)	14.0 (7.7-20.4)	13.3 (9.9-16.7)	.19	.94
Education level[e]							
Less than high school	12.7 (8.2-17.3)	10.1 (5.5-14.7)	8.7 (5.2-12.2)	9.0 (5.7-12.4)	12.0 (6.1-17.9)	.70	.66
High School or Equivalent	8.6 (4.3-12.8)	9.5 (5.5-13.6)	14.5 (9.5-19.6)	13.5 (9.8-17.2)	11.2 (7.1-15.3)	.18	.77
Some college or more	10.6 (7.5-13.8)	17.0 (13.9-20.1)	14.9 (11.6-18.3)	15.4 (12.6-18.1)	16.9 (12.8-21.0)	.10	.83

© 2023 Wang JS et al. *JAMA Network Open*.

Characteristic	Adults with MHO without use of medication for hypertension, hyperglycemia or high cholesterol, % (95% CI)[b]					*P* for trend[c]	
	**1999-2002 (n = 1073)**	**2003-2006 (n = 1198)**	**2007-2010 (n = 1725)**	**2011-2014 (n = 1625)**	**2015-2018 (n = 1765)**	**1999-2002**	**2003-2006**
Income-to-poverty ratio, %[f]							
≤100	11.9 (6.8-16.9)	11.6 (5.7-17.5)	9.0 (3.3-14.6)	12.7 (8.7-16.6)	12.2 (8.9-15.5)	.66	.67
101-399	10.5 (7.5-13.6)	13.4 (10.8-16.0)	13.2 (9.6-16.7)	13.8 (10.6-17.1)	13.5 (10.1-16.9)	.32	.96
≥400	8.8 (4.4-13.3)	14.8 (10.7-19.0)	15.8 (10.5-21.1)	16.9 (12.5-21.3)	18.6 (12.6-24.7)	.02	.31
Home ownership [g]							
Owned home	11.1 (8.4-13.9)	14.5 (11.7-17.2)	12.8 (9.8-15.7)	15.0 (12.3-17.7)	15.7 (11.6-19.7)	.08	.36
Rented home or other arrangement	8.8 (5.0-12.6)	11.8 (8.2-15.3)	14.3 (10.0-18.7)	12.5 (9.5-15.4)	13.3 (10.3-16.3)	.11	.91
Health insurance type [h]							
Private	9.8 (7.7-11.9)	14.4 (11.2-17.7)	15.1 (12.3-17.9)	14.6 (11.3-17.9)	16.5 (12.7-20.2)	.01	.44
Government	8.5 (1.2-15.7)	9.3 (4.4-14.2)	6.2 (2.7-9.8)	11.2 (6.4-16.0)	12.8 (9.0-16.6)	.07	.05
None	15.9 (11.1-20.7)	13.2 (8.7-17.6)	13.3 (8.5-18.1)	17.4 (10.4-24.3)	14.5 (9.1-19.9)	.67	.49
Weight group (BMI range, kg/m²)							
Class I obesity (30.0-34.9)	11.9 (9.1-14.7)	17.2 (13.8-20.6)	16.1 (12.9-19.3)	17.6 (14.7-20.6)	18.3 (14.2-22.4)	.02	.53
Class II obesity (35.0-39.9)	8.6 (4.9-12.4)	13.6 (9.1-18.2)	12.4 (9.3-15.6)	11.6 (7.3-15.9)	13.0 (7.6-18.4)	.41	.94
Class III obesity (≥40.0)	7.5 (3.2-11.8)	3.8 (1.7-5.8)	7.1 (3.6-10.6)	6.4 (3.4-9.4)	8.2 (4.3-12.2)	.32	.08

Abbreviation: MHO, metabolically healthy obesity; CI, confidence interval; BMI, body mass index (calculated as weight in kilograms divided by height in meters squared).

© 2023 Wang JS et al. *JAMA Network Open*.

[a] Individuals with self-reported use of medication for hypertension, hyperglycemia or high cholesterol were not considered as MHO; Estimates by age groups were unadjusted. Other estimates were age-standardized to the nonpregnant adult population with obesity in the 2015-2018 National Health and Nutrition Examination Survey cycles, using 3 age groups (20-39, 40-59, and ≥60 years) by the direct method.

[b] Sample sizes are unweighted. Data are presented as weighted percentage (95% CI).

[c] Trends over time from year ranges listed to 2015-2018 were evaluated using logistic regression.

[d] Self-reported according to fixed categories and classified as Mexican American, non-Hispanic Black, non-Hispanic White or other race and ethnicity (eg, non-Hispanic Asian or multiple).

[e] Missing for 3 participants (0%).

[f] Missing for 651 participants (6.7%).

[g] Missing for 77 participants (0.8%).

[h] Missing for 44 participants (0.4%).

© 2023 Wang JS et al. *JAMA Network Open*.

eTable 6. Trends in the Percentage of US Adults Without Any Metabolic Abnormalities or Previous Diagnosis of Cardiovascular Disease Among the Population With Obesity, 1999-2018[a]

**Characteristic**	**Adults without any metabolic abnormalities or previous diagnosis of CVDs, % (95% CI)[b]**					***P* for trend[c]**	
	**1999-2002 (n = 1064)**	**2003-2006 (n = 1193)**	**2007-2010 (n = 1715)**	**2011-2014 (n = 1621)**	**2015-2018 (n = 1757)**	**1999-2002**	**2003-2006**
Overall percentage	10.3 (8.5-12.0)	13.3 (11.0-15.6)	14.0 (11.4-16.5)	14.4 (12.6-16.2)	14.8 (12.1-17.4)	.01	.42
Age, y							
20-39	18.2 (12.9-23.4)	26.8 (20.8-32.7)	24.9 (18.7-31.1)	24.4 (19.7-29.2)	27.1 (21.6-32.7)	.10	.88
40-59	9.9 (6.3-13.4)	9.8 (6.1-13.4)	12.6 (9.4-15.7)	12.1 (8.8-15.4)	11.1 (7.3-14.9)	.45	.70
≥60	2.0 (0.1-3.8)	2.9 (0.9-4.9)	3.6 (1.4-5.7)	6.2 (3.7-8.7)	5.6 (2.6-8.6)	.01	.07
Sex							
Men	7.8 (5.1-10.5)	11.4 (7.5-15.2)	12.9 (9.5-16.3)	12.5 (9.9-15.2)	13.8 (10.2-17.5)	.03	.42
Women	12.3 (9.7-15.0)	15.3 (12.3-18.2)	14.9 (11.8-17.9)	15.8 (13.8-17.9)	15.6 (12.0-19.2)	.19	.77
Race and ethnicity[d]							
Mexican American	10.3 (7.2-13.5)	12.9 (8.4-17.4)	13.0 (9.1-16.9)	12.2 (8.6-15.9)	12.2 (8.8-15.7)	.88	.88
Non-Hispanic Black	13.4 (9.2-17.6)	18.8 (14.0-23.5)	16.7 (12.8-20.6)	15.9 (12.7-19.1)	15.1 (12.5-17.7)	.66	.10
Non-Hispanic White	7.2 (5.1-9.3)	11.9 (8.5-15.3)	13.4 (9.9-17.0)	14.1 (11.6-16.5)	15.6 (11.4-19.8)	.001	.15
Other	21.8 (12.3-31.4)	12.1 (4.1-20.0)	12.0 (7.0-17.0)	14.0 (7.2-20.7)	13.2 (9.6-16.8)	.26	.72
Education level[e]							
Less than high school	12.3 (7.8-16.7)	9.5 (5.4-13.5)	8.1 (4.8-11.4)	10.3 (6.3-14.2)	11.9 (6.3-17.6)	.99	.44
High School or Equivalent	8.2 (4.1-12.4)	9.6 (5.5-13.6)	14.9 (9.9-19.9)	13.7 (10.0-17.5)	10.9 (6.6-15.3)	.19	.88
Some college or more	10.8 (7.7-13.9)	16.6 (13.7-19.5)	15.7 (12.4-19.0)	15.9 (13.4-18.5)	16.9 (13.0-20.9)	.09	.81
Income-to-poverty ratio, %[f]							

© 2023 Wang JS et al. *JAMA Network Open*.

Characteristic	Adults without any metabolic abnormalities or previous diagnosis of CVDs, % (95% CI)[b]					*P* for trend[c]	
	1999-2002 (n = 1064)	2003-2006 (n = 1193)	2007-2010 (n = 1715)	2011-2014 (n = 1621)	2015-2018 (n = 1757)	1999-2002	2003-2006
≤100	12.1 (7.6-16.6)	13.9 (6.7-21.1)	8.2 (2.8-13.7)	12.6 (8.5-16.6)	11.0 (7.4-14.6)	> .99	.99
101-399	10.1 (7.1-13.1)	12.5 (9.8-15.2)	13.3 (9.8-16.9)	14.3 (11.3-17.3)	13.6 (10.3-17.0)	.15	.70
≥400	9.0 (4.4-13.5)	14.5 (10.7-18.3)	16.9 (11.7-22.1)	18.0 (13.1-22.9)	18.9 (12.9-24.9)	.02	.31
Home ownership[g]							
Owned home	10.9 (8.2-13.7)	14.1 (11.3-16.8)	13.3 (10.2-16.3)	15.6 (13.0-18.3)	15.7 (11.6-19.7)	.04	.30
Rented home or other arrangement	9.1 (5.2-12.9)	11.4 (7.9-14.9)	14.2 (9.9-18.6)	12.8 (9.4-16.2)	13.4 (10.4-16.4)	.12	.90
Health insurance type[h]							
Private	9.7 (7.6-11.8)	14.1 (11.1-17.2)	15.8 (12.9-18.7)	15.0 (11.9-18.1)	16.5 (12.7-20.4)	.007	.42
Government	8.3 (0.9-15.6)	9.1 (4.3-13.9)	6.0 (2.5-9.5)	12.7 (7.5-17.9)	12.8 (9.1-16.4)	.05	.03
None	15.4 (10.5-20.3)	12.7 (8.5-16.9)	13.2 (8.6-17.9)	17.4 (10.5-24.4)	13.6 (8.4-18.9)	.66	.45
Weight group (BMI range, kg/m²)							
Class I obesity (30.0-34.9)	11.7 (9.1-14.3)	16.8 (13.4-20.2)	16.6 (13.5-19.7)	18.3 (15.5-21.2)	18.4 (14.4-22.4)	.01	.43
Class II obesity (35.0-39.9)	8.9 (5.1-12.6)	13.2 (8.0-18.3)	12.5 (9.3-15.6)	12.3 (8.2-16.5)	13.0 (7.6-18.4)	.39	.98
Class III obesity (≥40.0)	7.2 (2.9-11.5)	3.8 (1.7-5.8)	7.9 (4.1-11.7)	6.4 (3.4-9.4)	7.9 (3.9-11.9)	.38	.15

Abbreviation: CVDs, cardiovascular diseases; CI, confidence interval; BMI, body mass index (calculated as weight in kilograms divided by height in meters squared).

[a] Estimates by age groups were unadjusted. Other estimates were age-standardized to the nonpregnant adult population with obesity in the 2015-2018 National Health and Nutrition Examination Survey cycles, using 3 age groups (20-39, 40-59, and ≥60 years) by the direct method. CVDs included congestive heart failure, coronary heart disease, heart attack, and stroke. 36 participants without information of CVDs history were further excluded from the main analyses (n=7386).

[b] Sample sizes are unweighted. Data are presented as weighted percentage (95% CI).

© 2023 Wang JS et al. *JAMA Network Open*.

[c] Trends over time from year ranges listed to 2015-2018 were evaluated using logistic regression.

[d] Self-reported according to fixed categories and classified as Mexican American, non-Hispanic Black, non-Hispanic White or other race and ethnicity (eg, non-Hispanic Asian or multiple).

[e] Missing for 3 participants (0%).

[f] Missing for 651 participants (6.7%).

[g] Missing for 77 participants (0.8%).

[h] Missing for 44 participants (0.4%).

© 2023 Wang JS et al. *JAMA Network Open*.

eTable 7. Trends in the Percentage of US Adults With Metabolically Healthy Abdominal Obesity Among the Population With Abdominal Obesity, 1999-2018[a]

Characteristic	Adults with metabolically healthy abdominal obesity, % (95% CI)[b]					*P* for trend[c]	
	1999-2002 (n = 1797)	2003-2006 (n = 1894)	2007-2010 (n = 2646)	2011-2014 (n = 2370)	2015-2018 (n = 2478)	1999-2002	2003-2006
Overall percentage	11.2 (9.4-13.1)	17.0 (14.8-19.1)	17.4 (14.8-20.1)	18.7 (16.8-20.6)	18.1 (15.3-20.9)	<.001	.43
Age, y							
20-39	20.3 (15.2-25.5)	31.5 (26.4-36.6)	30.5 (24.2-36.7)	32.9 (28.7-37.2)	30.4 (24.5-36.3)	.04	.91
40-59	12.1 (9.2-15.1)	14.8 (11.5-18.2)	18.3 (15.0-21.6)	17.2 (13.5-21.0)	16.4 (11.9-20.8)	.09	.71
≥60	2.8 (1.3-4.2)	7.4 (4.8-10.0)	5.6 (4.1-7.1)	8.6 (5.8-11.4)	9.9 (6.7-13.1)	<.001	.07
Sex							
Men	8.1 (5.6-10.7)	12.2 (8.8-15.7)	12.2 (9.0-15.4)	13.2 (10.3-16.1)	13.7 (9.9-17.5)	.03	.51
Women	13.4 (10.8-16.1)	20.4 (17.9-22.8)	20.9 (17.9-23.9)	22.1 (19.8-24.4)	21.1 (17.5-24.6)	.002	.68
Race and ethnicity[d]							
Mexican American	11.1 (8.7-13.4)	16.1 (12.0-20.2)	15.7 (11.6-19.8)	14.5 (8.7-20.3)	14.5 (11.5-17.5)	.64	.59
Non-Hispanic Black	14.6 (11.4-17.8)	18.3 (13.2-23.4)	16.8 (13.0-20.6)	16.8 (13.9-19.7)	16.5 (12.9-20.1)	.75	.59
Non-Hispanic White	10.2 (8.0-12.3)	17.3 (14.3-20.3)	18.0 (14.3-21.7)	19.9 (17.6-22.2)	19.3 (15.4-23.2)	<.001	.27
Other	11.6 (5.1-18.1)	13.1 (6.6-19.6)	14.3 (10.6-18.1)	16.7 (12.2-21.2)	15.1 (11.7-18.6)	.23	.34
Education level[e]							
Less than high school	9.6 (6.1-13.2)	10.3 (6.5-14.0)	12.7 (9.4-16.0)	10.6 (7.3-13.8)	13.6 (10.0-17.2)	.15	.41
High School or Equivalent	10.9 (7.1-14.7)	14.5 (10.9-18.0)	16.0 (11.7-20.3)	17.3 (13.3-21.4)	14.8 (9.4-20.3)	.15	.83
Some college or more	12.3 (9.2-15.4)	20.2 (17.3-23.2)	19.8 (16.4-23.2)	21.3 (18.8-23.8)	20.3 (16.6-24.1)	.01	.85
Income-to-poverty ratio, %[f]							

© 2023 Wang JS et al. *JAMA Network Open*.

Characteristic	Adults with metabolically healthy abdominal obesity, % (95% CI)[b]					*P* for trend [c]	
	1999-2002(n = 1797)	2003-2006 (n = 1894)	2007-2010 (n = 2646)	2011-2014 (n = 2370)	2015-2018 (n = 2478)	1999-2002	2003-2006
≤100	8.2 (4.7-11.8)	13.7 (8.3-19.0)	13.1 (8.4-17.7)	14.5 (10.1-18.8)	12.3 (9.0-15.7)	.16	.90
101-399	10.8 (8.7-12.9)	16.7 (13.9-19.4)	15.8 (11.7-19.8)	18.6 (16.3-20.8)	17.5 (14.1-20.9)	.005	.58
≥400	13.0 (8.9-17.2)	18.2 (14.7-21.7)	21.7 (17.8-25.6)	23.2 (19.4-27.1)	21.8 (15.8-27.8)	.006	.28
Home ownership[g]							
Owned home	13.0 (10.5-15.4)	17.1 (14.7-19.6)	17.6 (14.7-20.5)	20.9 (18.2-23.5)	19.8 (15.7-23.9)	.001	.11
Rented home or other arrangement	7.7 (4.6-10.8)	17.3 (13.3-21.2)	15.5 (12.0-18.9)	14.9 (11.2-18.5)	15.0 (12.3-17.6)	.04	.28
Health insurance type[h]							
Private	12.1 (10.0-14.3)	18.4 (16.0-20.9)	19.6 (16.8-22.4)	21.5 (19.1-23.9)	20.5 (16.8-24.2)	<.001	.26
Government	7.1 (2.1-12.1)	11.6 (7.5-15.7)	9.3 (6.7-11.9)	15.1 (10.6-19.5)	13.3 (11.1-15.6)	.008	.22
None	13.0 (8.3-17.8)	14.0 (10.1-18.0)	15.4 (11.1-19.7)	17.7 (11.8-23.6)	15.6 (11.4-19.8)	.37	.70
Weight group (BMI range, kg/m²)[i]							
Normal weight (18.5-24.9)	23.5 (13.4-33.6)	38.9 (30.2-47.6)	38.1 (26.5-49.7)	36.2 (25.2-47.3)	54.4 (39.7-69.0)	.001	.04
Overweight (25.0-29.9)	12.7 (9.3-16.1)	22.0 (17.5-26.5)	24.7 (20.4-29.0)	27.8 (23.6-31.9)	21.6 (17.1-26.2)	<.001	.71
Class I obesity (30.0-34.9)	10.9 (8.6-13.2)	15.9 (12.7-19.2)	14.5 (11.7-17.2)	17.3 (14.5-20.2)	17.5 (13.3-21.7)	.01	.42
Class II obesity (35.0-39.9)	8.6 (4.9-12.4)	13.8 (9.0-18.7)	11.7 (8.5-14.9)	11.2 (7.4-15.0)	12.2 (7.0-17.3)	.50	.86
Class III obesity (≥40.0)	6.7 (2.9-10.5)	3.2 (1.4-5.0)	7.3 (3.8-10.8)	6.2 (2.8-9.6)	7.6 (3.8-11.4)	.32	.07

Abbreviation: CI, Confidence Interval; BMI, body mass index (calculated as weight in kilograms divided by height in meters squared).

[a] Estimates by age groups were unadjusted. Other estimates were age-standardized to all nonpregnant adults with abdominal obesity in the 2015-2018 National Health and Nutrition Examination Survey cycles, using 3 age groups (20-39, 40-59, and ≥60 years) by the direct method. Abdominal obesity was defined as a waist circumference ≥ 102

© 2023 Wang JS et al. *JAMA Network Open*.

cm (men) or ≥ 88 cm (women). Metabolically healthy abdominal obesity was defined as having none of the four metabolic syndrome components among participants with abdominal obesity.

[b] Sample sizes are unweighted. Data are presented as weighted percentage (95% CI).

[c] Trends over time from year ranges listed to 2015-2018 were evaluated using logistic regression.

[d] Self-reported according to fixed categories and classified as Mexican American, non-Hispanic Black, non-Hispanic White or other race and ethnicity (eg, non-Hispanic Asian or multiple).

[e] Missing for 6 participants (0%).

[f] Missing for 984 participants (6.8%).

[g] Missing for 119 participants (0.8%).

[h] Missing for 66 participants (0.5%).

[i] Due to small sample size, estimates of the underweight group were not reported.

© 2023 Wang JS et al. *JAMA Network Open*.

eTable 8. Trends in the Prevalence of Metabolically Healthy Obesity (MHO) Among US Adults With Obesity by Different Criteria, 1999-2018[a]

Criteria	Adults with MHO, % (95% CI)[b]					*P* for trend[c]	
	1999-2002	2003-2006	2007-2010	2011-2014	2015-2018	1999-2002	2003-2006
Criteria 1							
Sample size	3554	3420	4583	4317	4161		
No. of adults with obesity	1058	1176	1681	1582	1706		
No. of MHO	360	457	624	652	675		
Prevalence of MHO	9.8 (8.6-11.0)	13.2 (11.6-14.7)	13.5 (11.8-15.2)	15.8 (14.3-17.4)	18.1 (16.1-20.2)	<.001	<.001
Proportion of MHO	33.6 (29.4-37.7)	38.6 (35.1-42.1)	38.6 (34.4-42.8)	42.5 (38.7-46.3)	42.4 (39.6-45.1)	<.001	.03
Criteria 2							
Sample size	3586	3468	4660	4424	4292		
No. of adults with obesity	1073	1198	1725	1625	1765		
No. of MHO	356	449	630	646	675		
Prevalence of MHO	9.6 (8.4-10.7)	12.9 (11.3-14.5)	13.4 (11.7-15.2)	15.5 (14.0-17.1)	17.8 (15.8-19.7)	<.001	<.001
Proportion of MHO	32.5 (28.5-36.6)	37.6 (34.0-41.2)	38.1 (33.7-42.4)	41.6 (37.9-45.2)	41.5 (39.0-44.1)	<.001	.03
Criteria 3							
Sample size	3656	3610	4794	4457	4391		
No. of adults with obesity	1108	1273	1774	1634	1801		
No. of MHO	228	236	238	149	147		
Prevalence of MHO	6.4 (5.7-7.1)	6.3 (5.0-7.6)	4.9 (4.1-5.7)	3.6 (2.7-4.4)	3.5 (2.8-4.2)	<.001	<.001
Proportion of MHO	21.7 (19.0-24.4)	18.3 (14.8-21.7)	14.3 (12.2-16.3)	9.7 (7.4-12.0)	8.5 (7.0-10.0)	<.001	<.001

© 2023 Wang JS et al. *JAMA Network Open*.

Criteria	Adults with MHO, % (95% CI)[b]					*P* for trend[c]	
	1999-2002	2003-2006	2007-2010	2011-2014	2015-2018	1999-2002	2003-2006
Criteria 4							
Sample size	3656	3610	4794	4457	4391		
No. of adults with obesity	1108	1273	1774	1634	1801		
No. of MHO	427	476	672	609	672		
Prevalence of MHO	12.1 (10.7-13.4)	13.2 (11.9-14.5)	14.3 (12.7-15.9)	14.8 (13.4-16.3)	16.7 (14.8-18.7)	<.001	.004
Proportion of MHO	41.3 (37.2-45.4)	38.8 (35.0-42.5)	41.0 (37.2-44.8)	40.0 (36.4-43.7)	40.2 (36.4-43.9)	.99	.64
Criteria 5[d]							
Sample size	3468	3353	4528	-	4210		
No. of adults with obesity	1024	1154	1667	-	1721		
No. of MHO	73	98	122	-	120		
Prevalence of MHO	2.3 (1.7-2.8)	3.2 (2.3-4.0)	2.9 (2.2-3.7)	-	3.5 (2.5-4.4)	.07	.59
Proportion of MHO	7.7 (5.7-9.6)	9.1 (6.9-11.3)	8.4 (6.5-10.2)	-	8.0 (6.0-9.9)	.77	.42
Criteria 6[d]							
Sample size	3576	3448	4615	-	4254		
No. of adults with obesity	1072	1196	1707	-	1744		
No. of MHO	305	379	533	-	558		
Prevalence of MHO	8.3 (7.4-9.3)	11.3 (9.6-12.9)	11.5 (9.9-13.2)	-	14.8 (12.9-16.7)	<.001	.002
Proportion of MHO	28.2 (25.0-31.5)	32.8 (28.9-36.8)	32.7 (28.7-36.7)	-	34.9 (31.6-38.1)	.02	.36

Abbreviation: CI, Confidence Interval.

© 2023 Wang JS et al. *JAMA Network Open*.

[a] The prevalence of adults with MHO was calculated relative to all nonpregnant adult participants, and age-standardized to the 2000 U.S. Census population, using 3 age groups (20-39, 40-59, and ≥60 years) by the direct method. The proportion of adults with MHO was calculated relative to only adults with obesity and age-standardized to the nonpregnant adult population with obesity in the 2015-2018 National Health and Nutrition Examination Survey cycles, using the same 3 age groups. Specific information of each definition of MHO are shown in eTable 2. Not all participants had all metabolic parameters measured; therefore, the sample sizes under some MHO criteria were not equal to the total number of participants for the survey cycles.

[b] Sample sizes and number of cases are unweighted. Prevalence estimates and proportion estimates are presented as weighted percentage (95% CI).

[c] Trends over time from year ranges listed to 2015-2018 were evaluated using logistic regression.

[d] The National Health and Nutrition Examination Survey did not collect laboratory values for CRP between 2011 and 2014; therefore, estimates for MHO under this definition in 2011-2014 cycle were not reported.

© 2023 Wang JS et al. *JAMA Network Open*.

eTable 9. Trends in Age-Adjusted Mean Concentrations of Individual Metabolic Parameters by Metabolic Health Status Among US Adults With Obesity, 1999-2018[a]

**Metabolic Parameter**	**Mean concentrations, % (95% CI)[b]**					***P* for trend[c]**	
	**1999-2002**	**2003-2006**	**2007-2010**	**2011-2014**	**2015-2018**	**1999-2002**	**2003-2006**
**Adults with Obesity**							
No.	1073	1198	1725	1625	1765		
BMI, kg/m^2	35.3 (34.8-35.8)	35.6 (35.3-36.0)	35.5 (35.3-35.8)	36.1 (35.6-36.6)	36.2 (35.8-36.7)	.002	.01
SBP, mm Hg	127.5 (126.5-128.4)	126.6 (125.5-127.8)	123.1 (122.2-124.0)	124.7 (123.7-125.6)	126.4 (125.5-127.3)	.16	.21
DBP, mm Hg	74.9 (73.8-76.0)	72.7 (71.7-73.7)	70.9 (70.0-71.9)	71.9 (71.2-72.6)	73.1 (72.1-74.0)	.06	.19
FPG, mg/dL	108.2 (105.6-110.7)	108.1 (106.1-110.0)	109.5 (107.4-111.7)	110.3 (107.5-113.1)	112.0 (110.0-114.0)	.002	.004
Triglycerides, mg/dL, geometric	144.9 (138.8-151.3)	142.7 (136.7-148.9)	128.2 (122.7-134.1)	125.1 (118.6-132.0)	111.2 (106.8-115.8)	<.001	<.001
HDL-C, mg/dL	46.2 (45.1-47.3)	49.3 (48.4-50.2)	48.1 (47.3-49.0)	48.2 (47.1-49.3)	49.1 (48.3-50.0)	.004	.87
waist circumference, cm	112.3 (110.9-113.7)	114.1 (113.2-114.9)	113.8 (113.1-114.6)	114.9 (113.7-116.1)	115.8 (114.7-116.9)	<.001	.005
HOMA, geometric	4.0 (3.8-4.3)	4.2 (4.0-4.4)	4.7 (4.6-4.9)	5.4 (5.1-5.7)	5.7 (5.5-5.9)	<.001	<.001
CRP, mg/L, geometric[d]	3.8 (3.5-4.2)	3.6 (3.4-3.9)	3.3 (3.2-3.4)	-	3.3 (3.0-3.6)	.03	.24
LDL-C, mg/dL	127.1 (124.4-129.8)	117.4 (114.8-120.1)	117.8 (115.5-120.1)	114.8 (112.9-116.7)	113.3 (111.1-115.6)	<.001	.006
**Adults with MUO**							
No.	960	1035	1511	1411	1534		
BMI, kg/m^2	35.4 (34.9-36.0)	36.0 (35.5-36.4)	35.8 (35.5-36.1)	36.5 (35.9-37.1)	36.6 (36.0-37.1)	.002	.03
SBP, mm Hg	128.5 (127.5-129.5)	127.9 (126.7-129.0)	124.2 (123.3-125.1)	126.3 (125.2-127.4)	127.9 (127.0-128.9)	.48	.09
DBP, mm Hg	75.5 (74.3-76.7)	73.5 (72.6-74.4)	71.7 (70.7-72.7)	72.8 (72.0-73.5)	73.8 (72.8-74.8)	.09	.19

© 2023 Wang JS et al. *JAMA Network Open*.

Metabolic Parameter	Mean concentrations, % (95% CI)[b]					*P* for trend[c]	
	1999-2002	2003-2006	2007-2010	2011-2014	2015-2018	1999-2002	2003-2006
FPG, mg/dL	109.7 (107.1-112.4)	109.9 (107.8-112.1)	111.9 (109.7-114.1)	113.0 (110.0-116.0)	114.9 (112.6-117.1)	<.001	.001
Triglycerides, mg/dL, geometric	154.4 (147.6-161.4)	154.0 (147.1-161.3)	136.6 (131.0-142.4)	134.3 (127.7-141.3)	119.2 (114.2-124.5)	<.001	<.001
HDL-C, mg/dL	44.8 (43.6-45.9)	47.8 (46.9-48.7)	46.6 (45.8-47.3)	46.4 (45.3-47.5)	47.4 (46.5-48.3)	.02	.85
waist circumference, cm	113.0 (111.6-114.4)	115.1 (114.0-116.1)	114.7 (113.9-115.6)	116.2 (114.9-117.5)	116.6 (115.3-117.9)	<.001	.02
HOMA, geometric	4.3 (4.0-4.5)	4.5 (4.3-4.7)	5.1 (4.9-5.3)	5.9 (5.5-6.2)	6.2 (6.0-6.5)	<.001	<.001
CRP, mg/L, geometric[d]	3.9 (3.6-4.3)	3.7 (3.4-4.0)	3.5 (3.3-3.6)	-	3.4 (3.0-3.9)	.07	.33
LDL-C, mg/dL	127.4 (124.5-130.4)	117.0 (114.3-119.7)	117.6 (115.0-120.2)	114.3 (112.2-116.4)	113.7 (111.3-116.0)	<.001	.03
**Adults with MHO**							
No.	113	163	214	214	231		
BMI, kg/m^2	33.7 (32.9-34.5)	34.3 (33.6-34.9)	33.6 (32.9-34.4)	34.2 (33.4-34.9)	34.6 (33.8-35.4)	.15	.03
SBP, mm Hg	118.2 (117.1-119.4)	116.0 (114.4-117.6)	115.3 (114.1-116.6)	114.3 (112.5-116.2)	115.8 (114.5-117.1)	.48	.54
DBP, mm Hg	71.2 (69.2-73.2)	69.4 (67.4-71.3)	68.1 (66.6-69.6)	67.5 (65.9-69.2)	69.4 (67.9-71.0)	.70	.31
FPG, mg/dL	90.6 (89.1-92.0)	92.5 (91.6-93.3)	92.0 (91.2-92.7)	90.8 (89.7-92.0)	91.6 (90.5-92.6)	.49	.04
Triglycerides, mg/dL, geometric [c]	89.1 (80.7-98.4)	91.0 (84.3-98.2)	93.2 (86.9-99.8)	83.0 (76.7-89.9)	75.6 (71.6-79.8)	<.001	<.001
HDL-C, mg/dL	59.8 (56.2-63.5)	57.5 (54.4-60.6)	56.2 (54.3-58.1)	59.1 (55.9-62.3)	59.1 (55.5-62.6)	.78	.76
waist circumference, cm	104.4 (102.3-106.6)	109.7 (106.9-112.4)	108.3 (106.5-110.1)	108.9 (106.4-111.3)	112.0 (110.2-113.8)	.002	.02
HOMA, geometric	2.2 (2.0-2.5)	2.9 (2.6-3.3)	3.1 (2.9-3.4)	3.2 (2.8-3.5)	3.2 (3.0-3.5)	<.001	.03
CRP, mg/L, geometric[d]	3.3 (2.6-4.1)	3.0 (2.2-3.9)	2.8 (2.3-3.5)	-	2.6 (2.2-3.0)	.47	.65

© 2023 Wang JS et al. *JAMA Network Open*.

Metabolic Parameter	Mean concentrations, % (95% CI)[b]					*P* for trend[c]	
	1999-2002	2003-2006	2007-2010	2011-2014	2015-2018	1999-2002	2003-2006
LDL-C, mg/dL	125.4 (115.0-135.9)	121.4 (115.2-127.6)	124.9 (118.1-131.7)	121.2 (117.4-125.1)	118.2 (114.0-122.4)	.01	.01

Abbreviation: CI, Confidence Interval; BMI, body mass index (calculated as weight in kilograms divided by height in meters squared); SBP, systolic blood pressure; DBP, diastolic blood pressure; FPG, fasting plasma glucose; HDL-C, high-density lipoprotein cholesterol; HOMA, the homeostasis model assessment; CRP, C-reactive protein; LDL-C, low-density lipoprotein cholesterol; MUO, metabolic unhealthy obesity; MHO, metabolically healthy obesity.

SI conversion factors: To convert FPG from mg/dL to mmol/L, multiply values by 0.0555; to convert TG from mg/dL to mmol/L, multiply values by 0.0113; to convert HDL-C, and LDL-C from mg/dL to mmol/L, multiply values by 0.0259.

[a] Estimated mean concentrations of individual metabolic parameters were age-standardized to the nonpregnant adult population with obesity in the 2015-2018 National Health and Nutrition Examination Survey cycles, using 3 age groups (20-39, 40-59, and ≥60 years) by the direct method. SBP, DBP, FPG, HDL-C, waist circumference and LDL-C were presented as arithmetic means; Triglycerides, HOMA and CRP were presented as geometric means due to the skewed distribution. Not all participants had all metabolic parameters measured; therefore, the sample sizes for some metabolic parameters were not equal to the total number of participants for the survey cycle.

[b] Sample sizes are unweighted. Data are presented as weighted mean (95% CI).

[c] Trends over time from year ranges listed to 2015-2018 were evaluated using linear regression.

[d] The National Health and Nutrition Examination Survey did not collect laboratory values for CRP between 2011 and 2014; therefore, estimates for CRP in 2011-2014 cycle were not reported.

© 2023 Wang JS et al. *JAMA Network Open*.

eFigure 1. Number of US Adults With Obesity, Metabolically Unhealthy Obesity (MUO), or Metabolically Healthy Obesity (MHO), 1999-2018

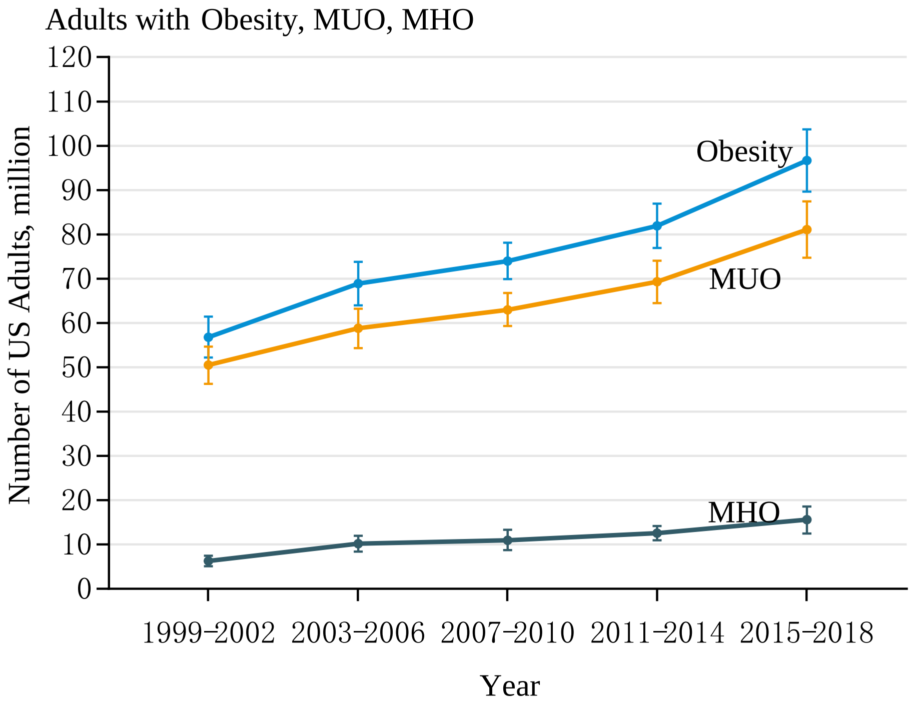

The number of US adults with obesity, MUO and MHO. Obesity was defined as a body mass index ≥ 30 kg/m^2. MUO was defined as having any component of the metabolic syndrome (waist circumference excluded) among participants with obesity. MHO was defined as meeting none of the metabolic syndrome criteria among participants with obesity. The numbers of individuals with obesity, MHO or MUO were calculated by multiplying the age-standardized prevalence estimates by the total noninstitutionalized adult population for each National Health and Nutrition Examination Survey cycle. All estimates were weighted and the error bars indicate 95% CIs.

© 2023 Wang JS et al. *JAMA Network Open*.

eFigure 2. Trends in the Prevalence and Proportion of Metabolically Healthy Obesity (MHO) Among US Adults With Obesity by Different Criteria, 1999-2018

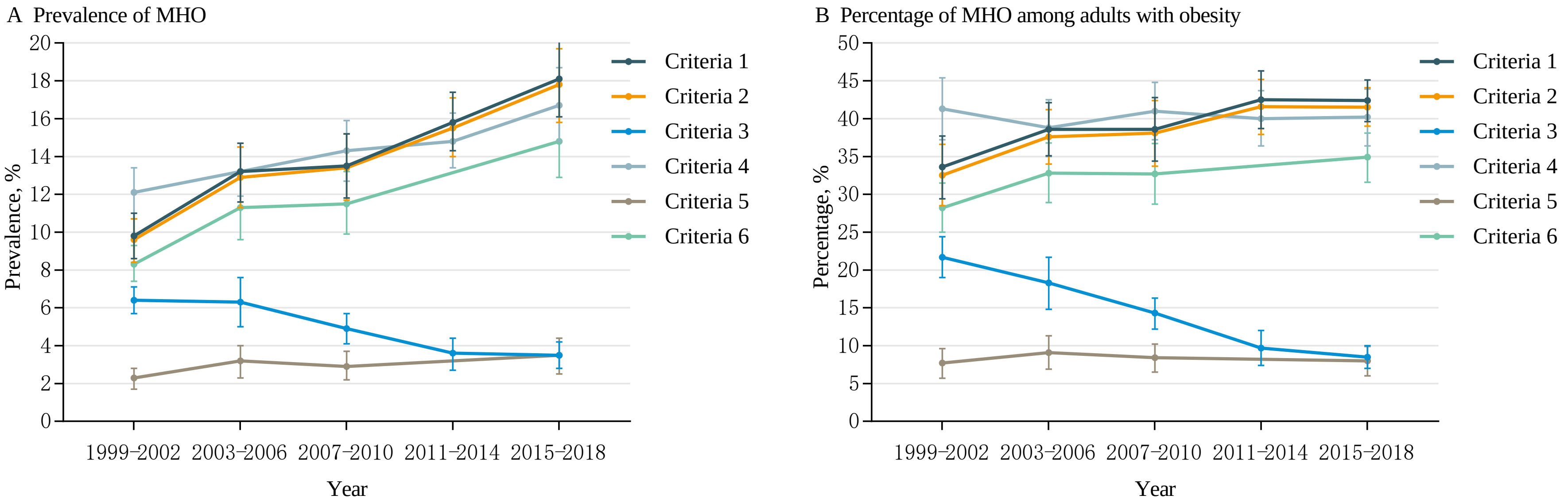

Trends in the prevalence of MHO among US adults (A), and its proportion among adults with obesity (B) by different criteria. Specific information of each definition of MHO are shown in eTable 2. The prevalence estimates in panel (A) were age-standardized to the 2000 U.S. Census population, using 3 age groups (20-39, 40-59, and ≥60 years) by the direct method. The proportion estimates in panel (B) were age-standardized to the nonpregnant adult population with obesity in the 2015-2018 National Health and Nutrition Examination Survey cycles, using the same 3 age groups. All estimates were weighted and the error bars indicate 95% CIs. The National Health and Nutrition Examination Survey did not collect laboratory values for C-reactive protein between 2011 and 2014; therefore, estimates for MHO under criteria 5 and criteria 6 in 2011-2014 cycle were not reported. Specific estimates are shown in eTable 8.

© 2023 Wang JS et al. *JAMA Network Open*.

eReferences

1. National Center for Health Statistics. NHANES survey methods and analytic guidelines. Centers for Disease Control and Prevention. Accessed April 1, 2022. https://wwwn.cdc.gov/nchs/nhanes/analyticguidelines.aspx
2. Executive Summary of The Third Report of The National Cholesterol Education Program (NCEP) Expert Panel on Detection, Evaluation, And Treatment of High Blood Cholesterol In Adults (Adult Treatment Panel III). *JAMA*. 2001;285(19):2486-2497
3. Alberti KG, Eckel RH, Grundy SM et al. Harmonizing the metabolic syndrome: a joint interim statement of the International Diabetes Federation Task Force on Epidemiology and Prevention; National Heart, Lung, and Blood Institute; American Heart Association; World Heart Federation; International Atherosclerosis Society; and International Association for the Study of Obesity. *Circulation*. 2009;120(16):1640-1645
4. Meigs JB, Wilson PWF, Fox CS et al. Body Mass Index, Metabolic Syndrome, and Risk of Type 2 Diabetes or Cardiovascular Disease. *The Journal of Clinical Endocrinology & Metabolism*. 2006;91(8):2906-2912
5. Kuk JL, Ardern CI. Are Metabolically Normal but Obese Individuals at Lower Risk for All-Cause Mortality? *Diabetes Care*. 2009;32(12):2297-2299
6. Karelis AD, Rabasa-Lhoret R. Inclusion of C-reactive protein in the identification of metabolically healthy but obese (MHO) individuals. *Diabetes Metab*. 2008;34(2):183-184
7. Wildman RP, Muntner P, Reynolds K et al. The obese without cardiometabolic risk factor clustering and the normal weight with cardiometabolic risk factor clustering - Prevalence and correlates of 2 phenotypes among the US population (NHANES 1999-2004). *ARCHIVES OF INTERNAL MEDICINE*. 2008;168(15):1617-1624
8. Matthews DR, Hosker JP, Rudenski AS et al. Homeostasis model assessment: insulin resistance and beta-cell function from fasting plasma glucose and insulin concentrations in man. *Diabetologia*. 1985;28(7):412-419

© 2023 Wang JS et al. *JAMA Network Open*.
