# Supplementary material for: Trends in the Prevalence of Metabolically Healthy Obesity Among US Adults, 1999-2018
Source: JAMA Netw Open. 2023 Mar 9;6(3):e232145. doi: 10.1001/jamanetworkopen.2023.2145 (PMC9999245; doi:10.1001/jamanetworkopen.2023.2145)
Supplement: Supplement 2. — Data Sharing Statement [file jamanetwopen-e232145-s002.pdf]

Data Sharing Statement

Wang. Trends in the Prevalence of Metabolically Healthy Obesity Among US Adults, 1999-2018. *JAMA Netw Open*. Published March 09, 2023. doi:10.1001/jamanetworkopen.2023.2145

Data
Data available: Yes
Data types: Deidentified participant data
How to access data: All data used for these analyses are publicly accessible on the NHANES website: https://wwwn.cdc.gov/nchs/nhanes/Default.aspx
When available: With publication

Supporting Documents
Document types: None

Additional Information
Who can access the data: Publicly available
Types of analyses: Since NHANES is public available, the data are always available for any project that requires it.
Mechanisms of data availability: The data are always publicly accessible on the NHANES website and can be accessed at any time.
Any additional restrictions: None
